# Phylogenomic and comparative analysis of the distribution and regulatory patterns of TPP riboswitches in fungi

**DOI:** 10.1038/s41598-018-23900-7

**Published:** 2018-04-03

**Authors:** Sumit Mukherjee, Matan Drory Retwitzer, Danny Barash, Supratim Sengupta

**Affiliations:** 1 Department of Physical Sciences, Indian Institute of Science Education and Research Kolkata, Mohanpur, 741246 India; 2 Department of Computer Science, Ben-Gurion University, Beer-Sheva, 84105 Israel

## Abstract

Riboswitches are metabolite or ion sensing cis-regulatory elements that regulate the expression of the associated genes involved in biosynthesis or transport of the corresponding metabolite. Among the nearly 40 different classes of riboswitches discovered in bacteria so far, only the TPP riboswitch has also been found in algae, plants, and in fungi where their presence has been experimentally validated in a few instances. We analyzed all the available complete fungal and related genomes and identified TPP riboswitch-based regulation systems in 138 fungi and 15 oomycetes. We find that TPP riboswitches are most abundant in Ascomycota and Basidiomycota where they regulate TPP biosynthesis and/or transporter genes. Many of these transporter genes were found to contain conserved domains consistent with nucleoside, urea and amino acid transporter gene families. The genomic location of TPP riboswitches when correlated with the intron structure of the regulated genes enabled prediction of the precise regulation mechanism employed by each riboswitch. Our comprehensive analysis of TPP riboswitches in fungi provides insights about the phylogenomic distribution, regulatory patterns and functioning mechanisms of TPP riboswitches across diverse fungal species and provides a useful resource that will enhance the understanding of RNA-based gene regulation in eukaryotes.

## Introduction

Riboswitches are conserved non-coding structural RNA sensors that bind specific metabolites and regulate gene expression^1–4^. A riboswitch is mainly composed of two domains; a highly conserved aptamer domain, which is responsible for binding of metabolites, and the expression platform that changes conformation on metabolite binding and regulates the expression of associated genes^2^. Based on the type of metabolite that binds to the aptamer domain, riboswitches are classified into different classes. At present, nearly forty different classes of riboswitches have been discovered mainly in the different lineages of bacteria^5^, although the thiamin-pyrophosphate (TPP) riboswitch has been also found in several eukaryotic lineages such as fungi, plants and green algae where they regulate the expression of the TPP biosynthesis and transporter genes^4,6–11^. TPP is a coenzyme derived from vitamin B1, which is essential for all forms of life^12^. It is synthesized *de-novo* in bacteria, archaea, fungi, algae and plants^7–9,11^. TPP riboswitch aptamers are highly conserved across different lineages and it is the most widespread among all the riboswitch classes^5,6^. In bacteria, TPP riboswitch regulates gene expression via control of transcription termination or translation initiation^13–15^ while in eukaryotes it regulates gene expression via alternative splicing^7,16,17^. Splicing of the pre-mRNA initiated by high TPP concentration and consequent activation of the riboswitch leads to a truncated mature mRNA and non-functional protein product thereby preventing the subsequent steps in the TPP biosynthesis pathway from occurring.

TPP riboswitches were experimentally validated in few species of fungi where they are involved in splicing and regulate the expression of TPP biosynthesis^7,16^ and transporter^17^ genes. The discovery of new TPP riboswitches in fungi and related species was held up due to sampling bias as well as due to the lack of application of computational tools for new riboswitch detection. However, the ongoing JGI 1000 fungal genome project^18^ has provided a vast amount of fungal genome sequence data for different classes of fungi that can be mined to detect additional instances of fungal TPP riboswitches. To understand the patterns of TPP riboswitch-based gene regulation in the fungal world, it is essential to acquire a complete picture of TPP riboswitch distribution across all the fungal species. Comparative genomics analysis can yield insights into the regulatory role of riboswitches by revealing new patterns of riboswitch-based regulation and identify potential new functional roles of riboswitch-regulated genes in the transport of the corresponding ligand^19–24^. Detailed phylogenomic analysis of riboswitch distribution^25^ can also shed light on the evolutionary history of fungal TPP riboswitches and suggest possible points of origin of these regulatory elements.

In this study, we have analyzed the 192-complete fungal and 17 complete oomycetes genomes and found the presence of TPP riboswitch-based gene regulation system in 138 fungi and 15 oomycetes. By correlating the location of the TPP riboswitches with the genes they regulate, we can identify or annotate many TPP biosynthesis and transporter genes. While we found many instances of fungal TPP riboswitches in the introns of 5′ UTR region, we also found several TPP riboswitches regulating both biosynthesis and transporter genes in the intronic region between two exons. The riboswitch location when correlated with the intron-exon structure of the genes, the number and location of the splice sites enabled us to predict the regulation mechanism in many cases. The TPP riboswitches found in the introns of 5′UTR regions, as well as the intronic region between exons, are all involved in alternative splicing. However, the effects of alternative splicing depend on the location of the TPP riboswitches and the size of the intron where the riboswitch is embedded. We predict a new type of fungal TPP riboswitches that are located in the 5′UTR of single exonic genes whose mode of regulations does not involve alternative splicing. We were unable to identify the precise regulatory mechanism and hope that our analysis will motivate further experimental scrutiny of this type.

Our computational approach to detection of TPP riboswitches in fungi and oomycetes is based on the profile Hidden Markov Model (pHMM)^26,27^. pHMM’s are used to find related sequences containing specific sequence motifs by aligning with a profile generated from a multiple sequence alignment using probabilistic methods. Since only very few instances of TPP riboswitch have been experimentally validated in fungi, we have used validated bacterial TPP riboswitches to construct the TPP-specific pHMM. The requirement for TPP binding induced conformational change necessarily constrains the variability permissible in TPP riboswitch sequences. It is therefore not surprising that TPP riboswitches share similar primary sequence motifs across bacterial and eukaryotic domains. This feature was exploited in one of the earliest detection^6^ of fungal and plant TPP riboswitches that employed a sequence and structural pattern search using prokaryotic TPP riboswitches as benchmarks and provides additional justification for searching fungal and oomycetes TPP riboswitches using a pHMM constructed from bacterial TPP riboswitches. Our study provides the first comprehensive analysis of the distribution, evolution and regulatory patterns of TPP riboswitches in fungi and oomycetes.

## Results and Discussion

The gene-specific TPP riboswitches detected by the Riboswitch Scanner^27^ were mapped onto well-resolved phylogenetic trees to present the distribution of these riboswitches across all fully sequenced fungal genomes. We analyzed this distribution and classified all fungal TPP riboswitches into four types based on their mechanism of regulation. We describe below the important features of the distribution of TPP riboswitches and their regulatory mechanisms.

### Distribution of TPP riboswitches in fungi

Even though TPP riboswitch has motifs that are highly conserved across all three domains of life, the mechanisms of TPP riboswitch-based gene regulation mechanisms can vary significantly from prokaryotes to eukaryotes. Supplementary Data S1 provides the detailed information on the TPP riboswitches found in each of the fungal species.

**Figure 1:**
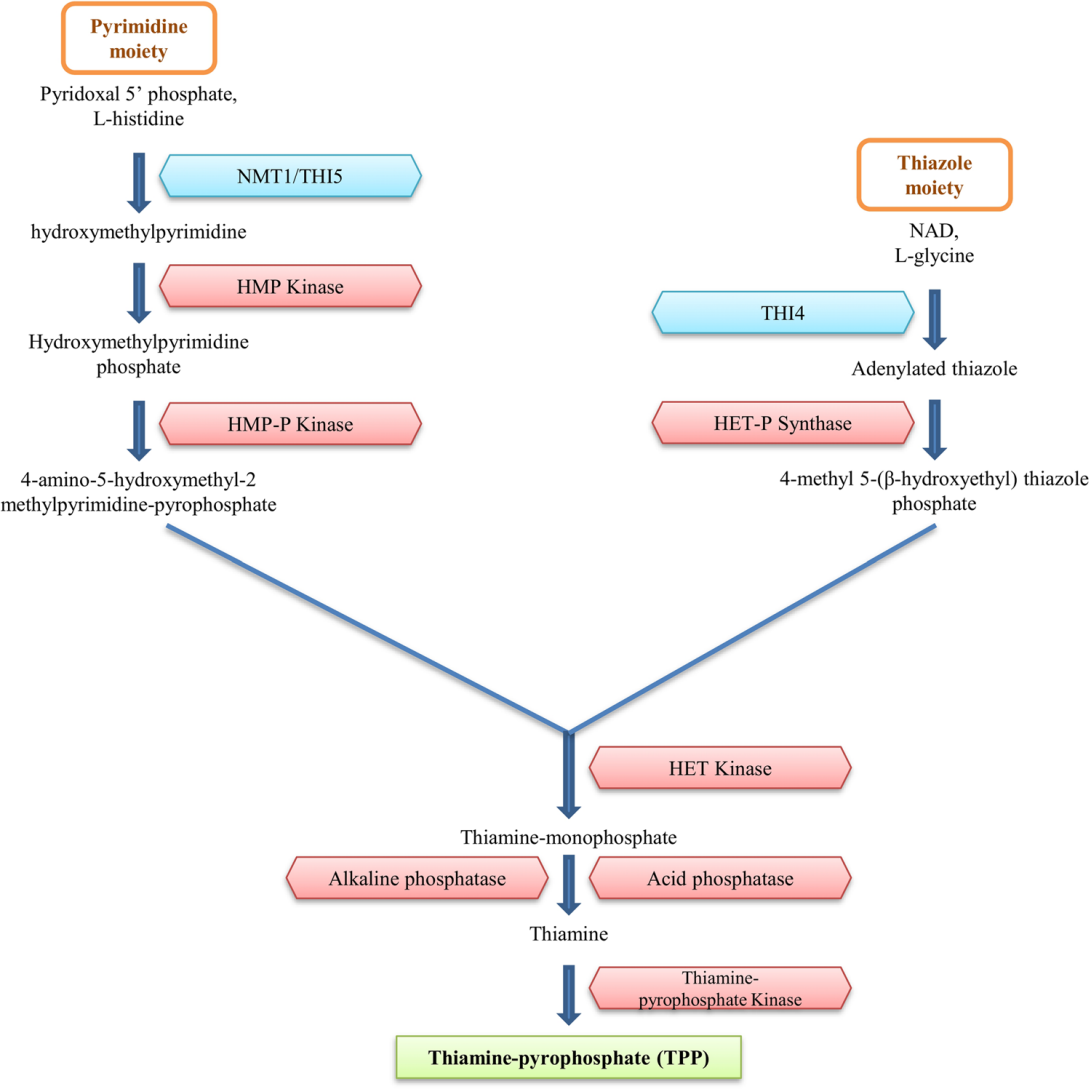
Fungal TPP biosynthesis pathway. TPP biosynthesis genes regulated by riboswitches are shown in blue. TPP biosynthesis genes that are not regulated by riboswitches in any organism are shown in red.

The TPP biosynthesis pathway is observed in most of the fungal species. The TPP biosynthesis pathway depicted in Fig. 1 is based on the presence of riboswitch-regulated TPP biosynthesis genes in different fungal species. The riboswitch-regulated genes are shown in blue. The biosynthesis genes for which there is no evidence of being regulated by a riboswitch in any species are shown in red. We found only two TPP biosynthesis genes NMT1 (hydroxymethylpyrimidine synthase) and THI4 (thiazole synthase) that are regulated by riboswitches. Both these genes are necessary to initiate the TPP biosynthesis pathway.

**Figure 2:**
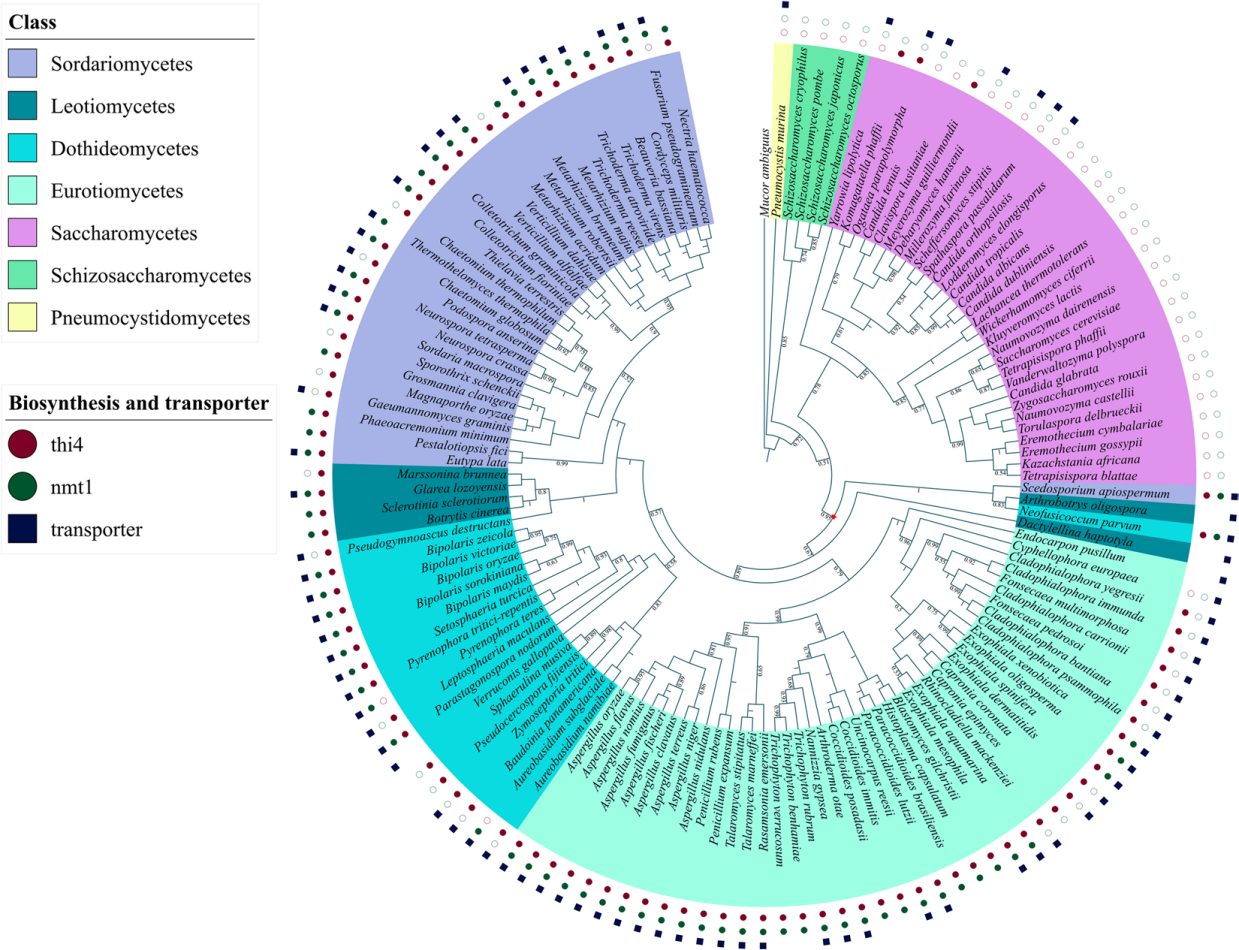
Phylogenomic distribution of riboswitch regulated TPP biosynthesis and transporter genes in the phylum Ascomycota. The different biosynthesis and transporter genes are color-coded according to a scheme specified in the figure legend. Filled circles and squares indicate both the riboswitch and the gene are present, unfilled circles and squares indicate that the riboswitch is absent but the corresponding gene is present. The absence of circle or square indicates that both the gene *and* riboswitch are missing in the corresponding organism.

**Figure 3:**
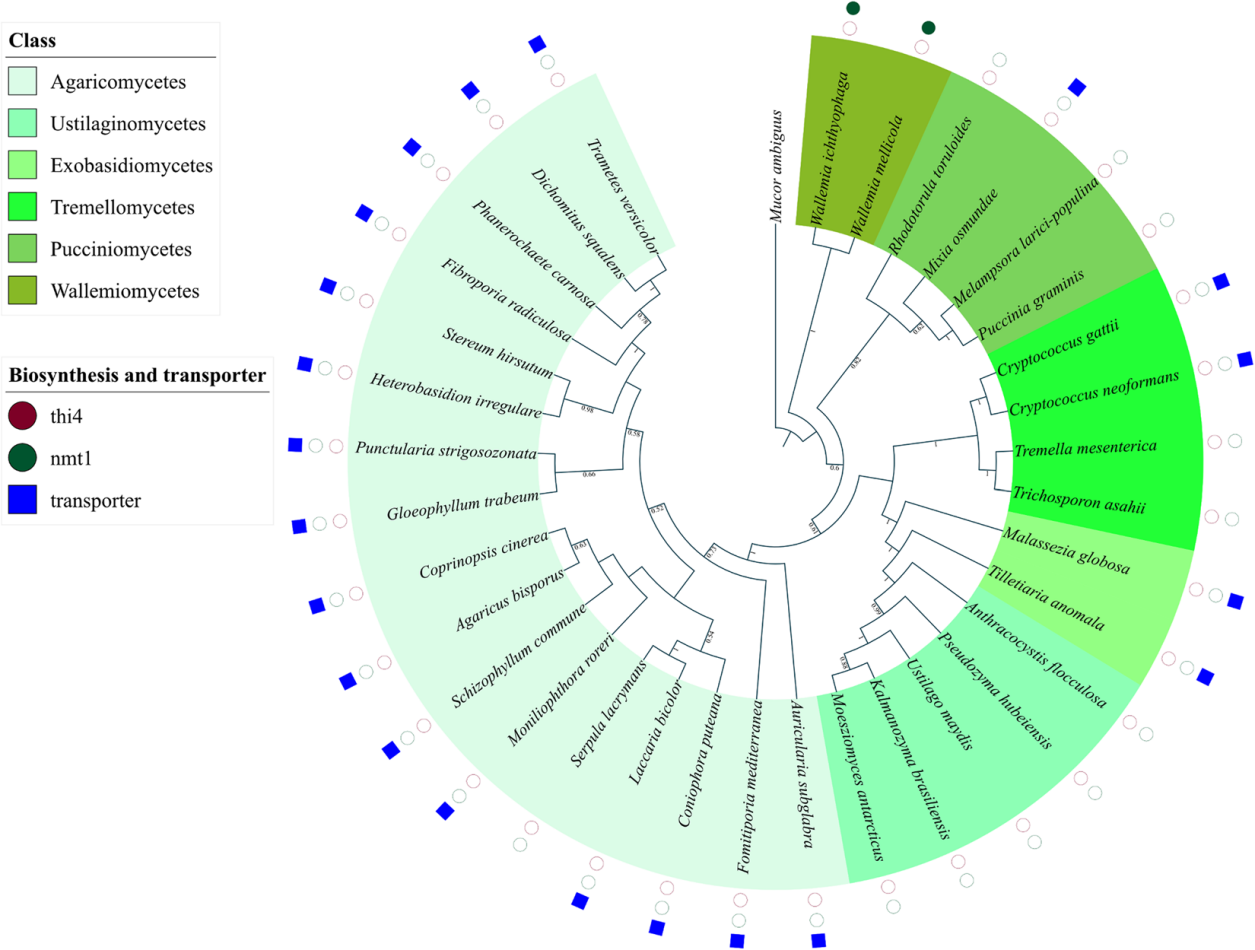
Phylogenomic distribution of riboswitch regulated TPP biosynthesis and transporter genes in the phylum Basidiomycota. The different biosynthesis and transporter genes are color-coded according to a scheme specified in the figure legend. Filled circles and squares indicate both the riboswitch and the gene are present, unfilled circles and squares indicate that the riboswitch is absent but the corresponding gene is present. The absence of a square indicates that both the transporter gene *and* riboswitch are missing in the corresponding organism.

TPP riboswitch-based gene regulation of biosynthesis genes is predominant in Ascomycota family, the largest phylum of the Fungi kingdom. Figure 2 shows the phylogenomic distribution of TPP riboswitches in Ascomycota family. Riboswitch regulation of TPP biosynthesis genes THI4 or NMT1 is ubiquitous across species belonging to all classes of Ascomycota except Saccharomycetes, Schizosaccharomycetes, and Pneumocystidomycetes. The remaining classes form a clade and the origin of riboswitches regulating these biosynthesis genes can be traced back to the point indicated by a red star in Fig. 2. In three species of this clade, the biosynthesis genes themselves are absent. However, the presence of TPP riboswitch regulating a transporter gene in these species indicates that these organisms can control TPP uptake from the environment. In Saccharomycetes, TPP riboswitches were found to regulate the THI4 biosynthesis gene in just three species as shown in Fig. 2. Outside of the Ascomycota family, we have found the presence of the riboswitch-regulated NMT1 gene in just three species two of which are depicted in Fig. 3. The third instance was found in a species belonging to the genus *Mucor* from the Zygomycota phylum (see Supplementary Data S2).

In addition to biosynthesis genes, we detected TPP riboswitches in various transporter genes that are distributed across different fungal phyla. The phylogenomic distribution of these TPP riboswitch regulated transporters is shown in Figs 2 and 3. Based on the presence of conserved domains in the protein sequences of such transporter genes, we have classified the fungal TPP riboswitch regulated transporter genes into three different orthologous classes. These are nucleoside, urea, and amino acid transporter families. (See methods section for specific criterion used in identifying conserved domains.) Classification of the transporters into different orthologous classes in fungi is therefore important in understanding the distribution of the regulatory mechanisms operational across those classes. In Ascomycota, we found several species having TPP riboswitches regulating the nucleoside transporter gene families. In almost all these cases, the regulatory mechanism is based on alternative splicing and relies on the presence of a stop codon either in an internal intron or at the end of an upstream Open Reading Frame (uORF). In multiple species where a nucleoside transporter is absent, a TPP riboswitch is found to regulate the transporter gene that belongs to the urea transporter family. These urea transporters are also found in the fungal phylum Basidiomycota and their regulatory mechanism is based on alternative splicing involving long-distance base pairing^17^. No other biosynthesis or transporter genes are regulated by this mechanism. The identification of a TPP riboswitch upstream to such generic transporters in both Ascomycota and Basidiomycota indicates that those transporters are likely to have some role in importing compounds related to TPP metabolism. We found another class of TPP riboswitch-regulated transporter genes that belong to amino-acid transporter families. These are widespread in Basidiomycota (see Fig. 3) but absent in Ascomycota. Analysis of Gene Ontology^28^ molecular function and NCBI-CDD (conserved domains database)^29^ searches revealed the presence of a highly conserved amino acid transporter domain in those proteins. The structure-based energy minimization method^30–33^ of detection provided further confirmation of the presence and role of TPP riboswitches in regulating the amino-acid transporter gene families in Basidiomycota. The presence of a riboswitch regulating different transporter gene families suggests that it is not possible to associate them exclusively with the transport of specific nucleosides, amino acids or urea. It is likely that those genes are multifunctional genes capable of transporting different types of ligands and their regulation by TPP riboswitches implicates them in the transport of ligands related to Thiamin.

An unusual exception to this pattern of regulation is found in *Gloeophyllum trabeum*, where the riboswitch is in the intron of the gene encoding eukaryotic translation initiation factor 5B. In *Postia placenta*, two copies of this gene encoding eukaryotic translation initiation factor 5B were found with the TPP riboswitch being in the 3′UTR of both these gene copies. Additional examination by BLAST^34^ reveals that these two copies share a high degree of similarity suggesting that the second copy of the gene along with the riboswitch appeared because of a gene-duplication event. The structure-based energy minimization method^30–33^ was not able to validate this finding because the presence of suboptimal solutions that are relatively close in energy to the optimal one. Therefore, further experimental studies based on this finding can provide fresh insight into translation inhibition due to the regulation of the initiation factor gene by the TPP riboswitch.

To understand the conserved structural features of fungal TPP riboswitches, we performed an analysis based on the alignment of the introns where the TPP riboswitch is located. We chose *Neurospora crassa* as the reference species and selected nine other random species from the different classes of fungi where a TPP riboswitch was found to regulate the same gene and extracted the full sequences of the introns. We highlighted the conserved base-pair stems (P1 to P5) on the multiple sequence alignment of the introns (Fig. S3-2, S3–4 and S3–6 of Supplementary Information). This analysis was carried out for three widely distributed fungal TPP riboswitch regulated genes THI4, NMT1, and urea transporter. In each of these cases, P4 and P5 stems were the most conserved regions of the fungal TPP aptamers across the phylogeny. As reported in a previous study^7^, the base-pairing between nucleotides in P4 and P5 stems and a complementary region adjacent to the proximal 5′ splice site are responsible for the regulation of NMT1 gene by alternative splicing. Hence, high level of conservation of P4 and P5 stems indicates their importance in splicing-based regulation in fungi. Even though the P1 and P2 stems of THI4 TPP riboswitch aptamers were fairly well conserved across the phylogeny significant variations were observed in nucleotides of these regions for the TPP aptamers associated with NMT1 and transporter genes. The P3 element of fungal TPP aptamers varies in both sequence and length. Many representatives of the fungal TPP aptamer possess an extended P3 stem while others have a shorter P3 stem.

**Figure 4:**
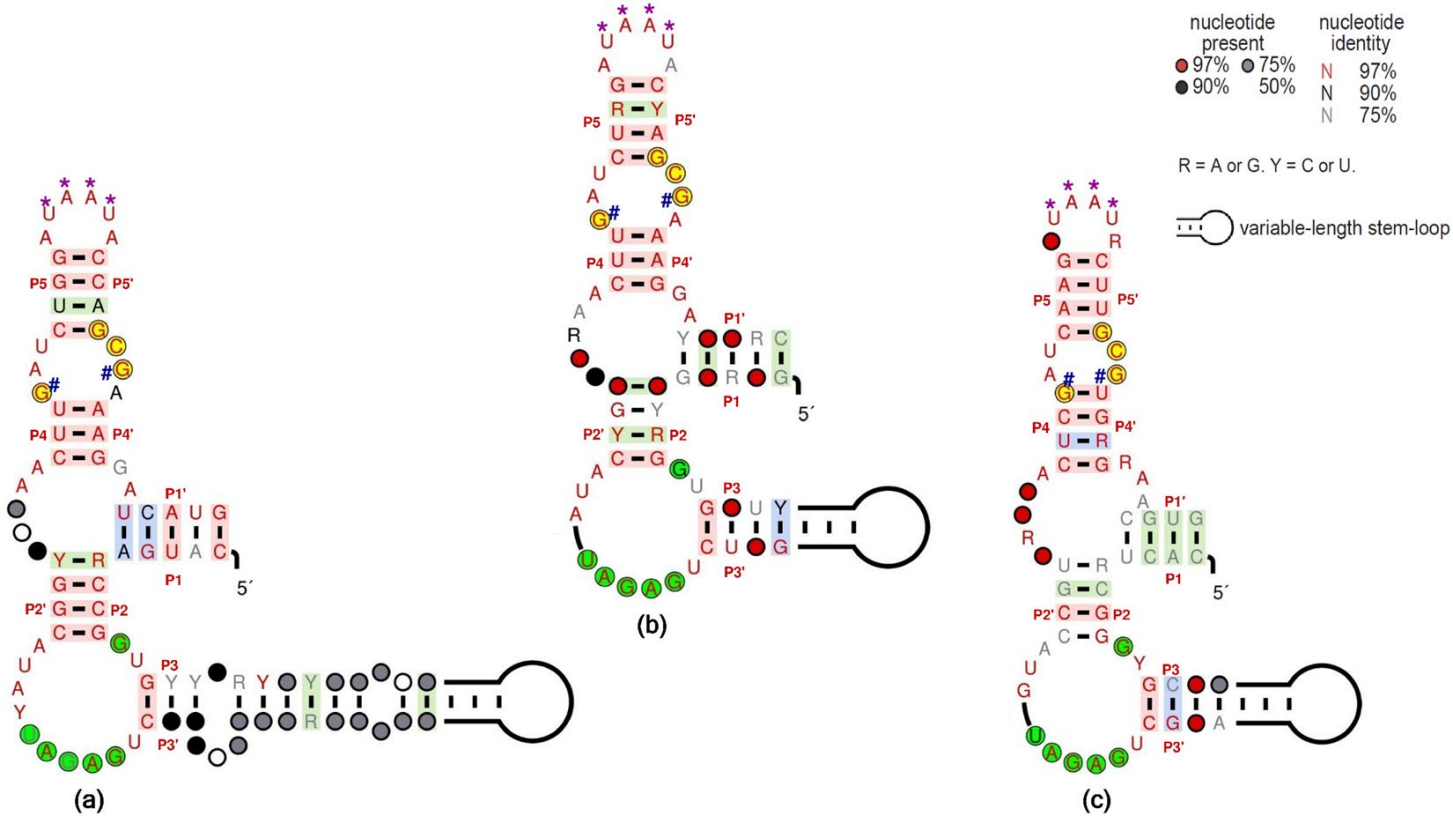
Consensus structures of fungal TPP aptamer involved in regulation of the (**a**) THI4 gene, (**b**) NMT1 gene and (**c**) urea transporter gene family. Bases that are responsible for pyrimidine recognitions are highlighted by green background and bases responsible for pyrophosphate recognitions are highlighted by yellow background. On TPP binding, the bases that form a non-canonical G-G base pair are marked with navy-blue hash marks. The conserved UAAU motif that is responsible of the complete closing of the two halves of the aptamer after initial binding of the aromatic ring to the GAGAU motif are marked with purple stars. The empty circles in the figure imply that most sequences in the alignment don’t have a specific nucleotide to fit those positions.

The well resolved crystal structures of TPP aptamers^35–38^ in the TPP bound state provide a detailed rendering of the residues involved in TPP binding and the changes in conformation that results on TPP binding. Certain motifs of the TPP aptamers identified from crystal structures of eukaryotic^35^ and prokaryotic^36,37^ TPP riboswitches are responsible for the recognition of pyrimidine and pyrophosphate and are found to be highly conserved across the phylogeny. We used the Rfam seed alignment (based on the covariance model) of TPP and the *cmsearch* application to search against a database consisting of the ten reference sequences mentioned above and generated separate sequence alignments of the TPP aptamers regulating THI4, NMT1 and urea transporter genes. R2R^39^ was then used to draw the consensus secondary structure of each of these TPP riboswitches. We mapped the conserved motifs and highlighted the important residues onto the consensus secondary structures (see Fig. 4) and the RNAFold-derived secondary structures of the TPP riboswitches in *Neurospora crassa* (see Fig. S3–1, S3-3, S3–5 of Supplementary Information). The GAGAU motif that recognizes the TPP aromatic ring has been highlighted in green and the conserved GCG motif in the junction J4/5 (loop between P4 and P5) that interacts with the pyrophosphate moiety is highlighted in yellow in Fig. 4 and Fig. S3 of the Supplementary information. The conserved UAAU motif in the loop L5 that is responsible of the complete closing of the two halves of the aptamer after initial binding of the aromatic ring to the GAGAU motif have been marked by purple stars in Fig. 4. The crystal structure^35^ also shows a non-canonical G-G base pair in junction J4/5 which is essential for pyrophosphate recognition (indicated by # marks in Fig. 4 and Fig. S3 of Supplementary Information) and the separation distance between these two G’s is conserved and corresponds to 18 bases. All such sequence motifs are highly conserved and found in all of the TPP aptamers associated with THI4, NMT1 and various transporter genes of fungi.

### TPP riboswitch-based gene regulations in Oomycetes

Oomycetes form a different phylogenetic lineage of fungus-like eukaryotic microorganisms. This group was originally classified among the fungi, based on common morphology and lifestyle^40^. Cladistic analysis^40^ based on recent discoveries about the biology of these organisms indicates a comparatively close relationship with some photosynthetic organisms, such as brown algae and diatoms. Taxonomic classification^41^ places the class oomycetes along with other classes such as Phaeophyceae (brown algae) within the phylum Heterokonta. Enzymatic steps of the TPP biosynthesis pathways of oomycetes are similar to fungi. In oomycetes, TPP riboswitches are found to regulate both TPP biosynthesis NMT1 and THI4 genes in a manner similar to fungi. Some species of the *Pythium* genus possess a riboswitch regulating the second biosynthesis pathway gene phosphomethylpyrimidine kinase (HMP-Kinase) instead of the first pathway gene (NMT1) as was the case in fungi. Another unusual feature found in the species belonging to the genus *Saprolegnia* was the presence of two TPP aptamers within the introns of 5′ UTR region of NMT1 gene. To further investigate the relation between these two TPP aptamers, we constructed a phylogenetic tree of all oomycetes riboswitch sequences based on the Neighbour-Joining (NJ)^42^ method. These two sequences having a high sequence similarity were found to cluster together in the phylogenetic tree thereby suggesting that one of them could plausibly have appeared after a duplication event. Earlier studies on bacterial riboswitches found two riboswitches in the 5′ UTR of *Bacillus clausii* metE mRNA. Those recognized two different metabolites and regulated transcription by mimicking a Boolean NOR logic gate^43,44^. But in the cases of *Saprolegnia*, we were unable to infer the functional mechanism employed by these two TPP aptamers in regulating the downstream NMT1 gene. Hence, further experimental studies are needed to explain this unique pattern of TPP riboswitch-based gene regulation in oomycetes.

TPP riboswitches are also found to regulate the transporter genes in oomycetes that can be broadly classified into two orthologous groups. The first belongs to the urea transporter family, which was found only in the two species, *Phytophthora cinnamomi*, and *Phytophthora sojae*. This class of transporter was also found in the fungi where they are regulated by alternative splicing involving long-distance base pairing. The second orthologous group of transporters is unique to oomycetes and is widely distributed across diverse species of oomycetes. The systematic functional annotation of those transporter genes reveals that they belong to the sodium symporter superfamilies. Supplementary Data S2 provides detailed information on the TPP riboswitches found in oomycetes.

### Regulatory mechanisms of TPP riboswitches in fungi and oomycetes

Mechanisms of riboswitch-controlled gene regulation vary from prokaryotes to eukaryotes. Regulation of TPP biosynthesis and transporter genes in bacteria is mainly carried out by either prematurely terminating transcription or by inhibiting translation. It was experimentally shown that in fungi, TPP riboswitch-based gene regulations are carried out via alternative splicing. Two mechanisms based on alternative splicing were experimentally uncovered in the fungi *Neurospora crassa*^7,17^. Based on the location of the TPP riboswitches, we have classified TPP riboswitch based gene regulation mechanisms in fungi into four types of which the first three modes of regulations are based on alternative splicing.

**Figure 5:**
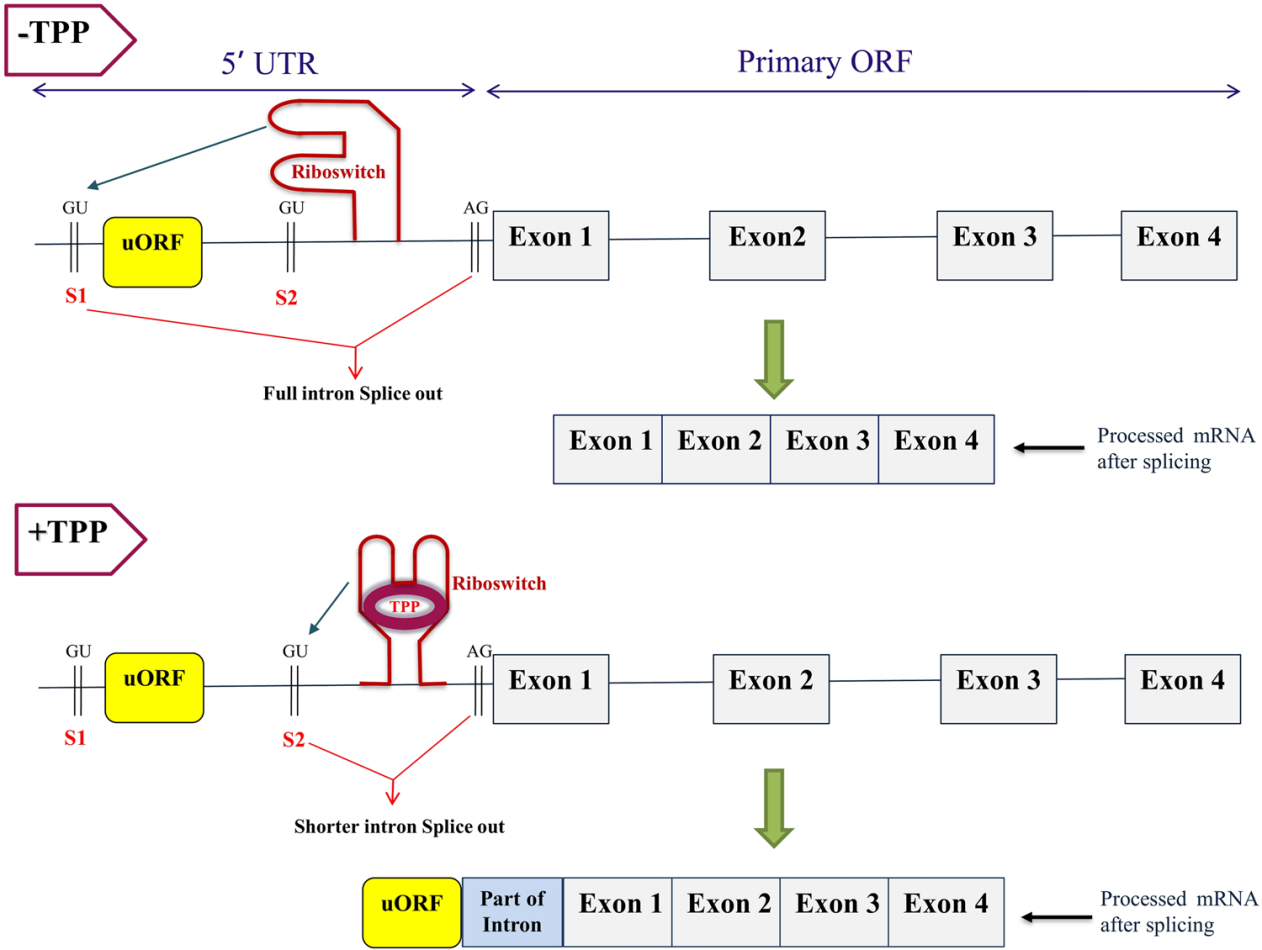
Generalized model of TPP riboswitch-based splicing (Type 1) observed when the riboswitch is located in the intron of the 5′UTR of the corresponding biosynthesis/transporter genes in fungi. The top panel of the figure represents the TPP-free state of the riboswitch, which promotes the interaction with the S1 site, and splicing of the full intron containing a short uORF. The bottom panel of the figure represents the TPP-bound state of the riboswitch, which promotes the interaction with the S2 site, and splicing of the shorter intron. The thin black line between successive exons represents the introns. The uORF is highlighted in yellow. The arrow pointing towards GU indicates activation of the corresponding splice site. The thick green arrows point to the resulting processed mRNA obtained after splicing.

The first type of splicing-based regulation (Type I) was discovered experimentally in *Neurospora crassa*^7^. In this type of regulation, the TPP riboswitch resides within the introns of 5′UTR. This type of regulations requires the presence of two alternative donor splice sites (GU), one upstream (S_1_) and another downstream (S_2_) to a short uORF in the intron of the 5′UTR region (see Fig. 5). When TPP concentration is low, the riboswitch segregates the alternative splice site S_2_, interacts with upstream donor splice site (S_1_) exposing it and enabling splicing of longer intron containing uORF, leading to the translation of the working TPP biosynthesis or transporter genes (see top panel of Fig. 5). At high TPP concentrations, the TPP-bound riboswitch changes its conformation to interact with the alternative donor splice site (S_2_), which promotes splicing of the shorter intron (see bottom panel of Fig. 5). This leads to translation of the uORF producing a short, non-functional poly-peptide fragment and preventing translation of a functional biosynthesis/transporter gene. We have found that this type of regulation controls synthesis of NMT1, THI4 and transporter genes in many fungi (Supplementary Data S1) and oomycetes (Supplementary Data S2), where the riboswitch is located in the intronic regions of 5′UTR. Such TPP riboswitches were detected by identifying the short uORF, the alternative splice sites S_1_ and S_2_ upstream and downstream to the uORF in the 5′ UTR and the splice site AG just before the start of Exon 1, downstream of the riboswitch sequence. Examples are provided in Fig. S4 of Supplementary Information.

**Figure 6:**
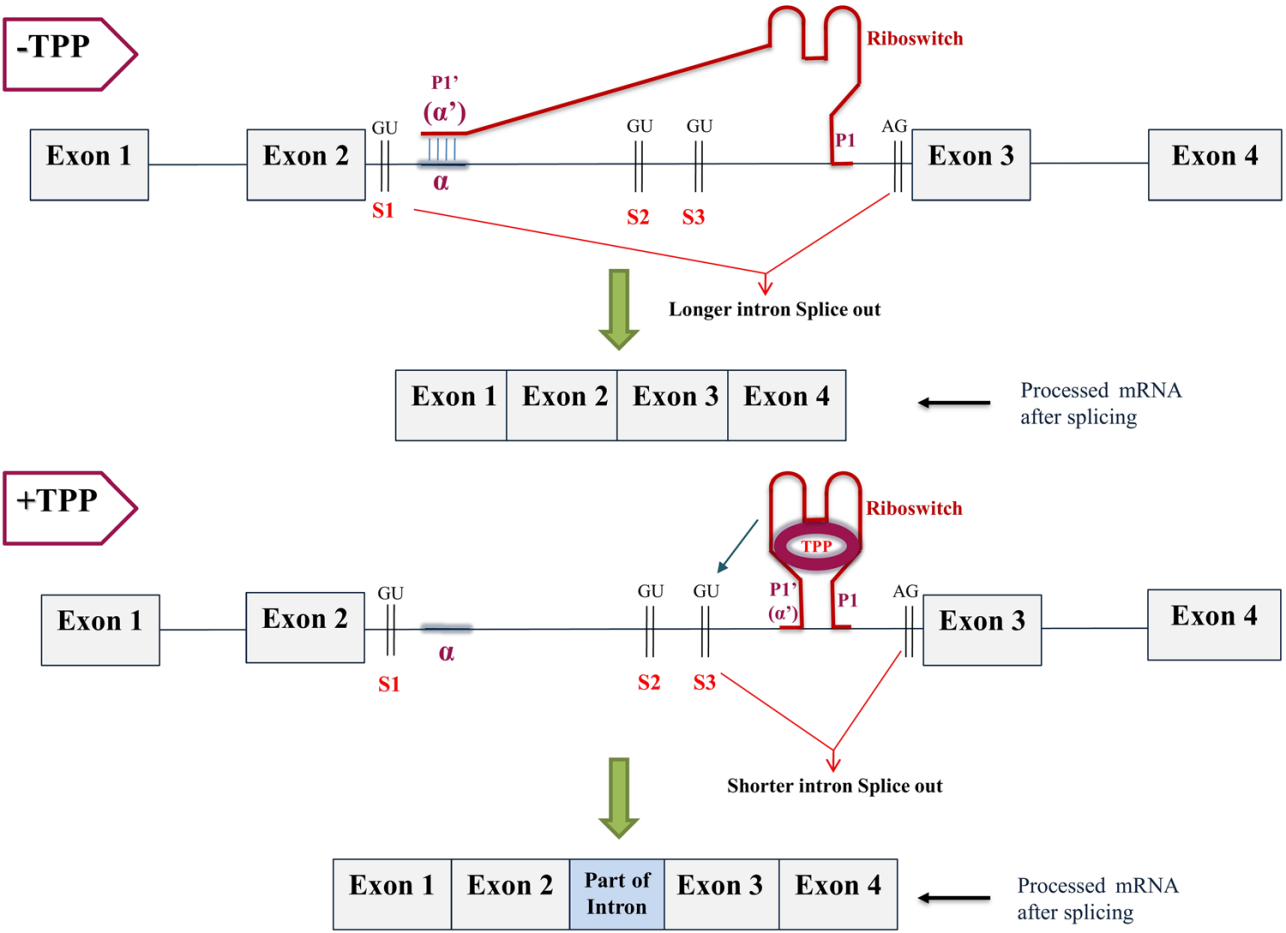
Generalized model of TPP riboswitch-based splicing (Type II) observed when the riboswitch is present in the large internal intron of the transporter genes in fungi and involved in long-distance base-pairing. The top panel of the figure represents the TPP-free state of the riboswitch which promotes the long-distance base pairing of the P1′ (α′) stem of the TPP aptamer with a distal α segment near the S1 site. Such an interaction promotes splicing of the larger intron. The bottom panel of the figure represents the TPP-bound state of the riboswitch where P1′ (α′) segment is incorporated into the aptamer forming a stable pairing with a complementary segment in the P1 stem. Such conformational changes promote interaction with alternative splice site and splicing of the shorter intron portion. The thin black line between successive exons represents the introns. The arrow pointing towards GU indicates activation of the corresponding splice site. The thick green arrows point to the resulting processed mRNA obtained after splicing.

The second type of TPP riboswitch mediated splicing-based regulation mechanisms (Type II) was experimentally detected in NCU01977 gene of *Neurospora crassa*^17^. In this type of regulation, TPP riboswitch is located in a long intron (having length 650–900 nucleotides) of the transporter genes that belong to urea transporter families. This mode of splicing^17^ involves long distance complementary base pairing interaction (~500 nucleotide separation) between a TPP aptamer segment and a conserved sequence element (α) located between the alternative 5′ splice sites S_1_ and S_2_. This mechanism, which requires the presence of at least two alternative donor splice sites, and a region (α) having the complementary sequence motif as the P1′ riboswitch stem. The central portion of the α element is highly conserved with the consensus sequence pattern 5′-RGCGGYRRY-3′; where R is a purine and Y is a pyrimidine, although the position of R and Y vary in some species of fungi) across different fungi. In this mechanism when the concentration of TPP is low, the segment (α′) near the 3′ end of the aptamer which is also the complementary to the sequence segment in the P1 stem complementary base pairs (see top panel of Fig. 6) with the distal conserved sequence element (α) located near the 5′ splice site (S_1_). This interaction promotes cutting out of longer intron and translation of working transporter gene (see top panel of Fig. 6 and Fig. 6 of Li and Breaker^17^). When the concentration of TPP is high, conformational changes lead to the α’ segment incorporated into the aptamer forming a stable pairing with a complementary segment in the P1 stem (see bottom panel of Fig. 6). This reduces splicing at the distal 5′ splice site (S1) and promotes splicing at one of the more proximal alternative 5′ splice sites (S2 and S3) which facilitate the reduced expression of the main ORF (see bottom panel of Fig. 6 and Fig. 6 of Li and Breaker^17^). TPP riboswitches of this type were identified by determining the size of the riboswitch-carrying intron, various alternative splice sites (S_1_, S_2_, S_3_ etc.) and the conserved sequence element (α) between the 5′ and 3′ splice sites and in the P1 stem. The comparative genomic analysis reveals that this type of regulation is observed in many fungal transporter genes belongs to the urea transporters families where the TPP riboswitch is present in the large internal intronic regions (see Supplementary Dataset S1 for detailed listings and Fig. S4 of Supplementary Information for examples).

**Figure 7:**
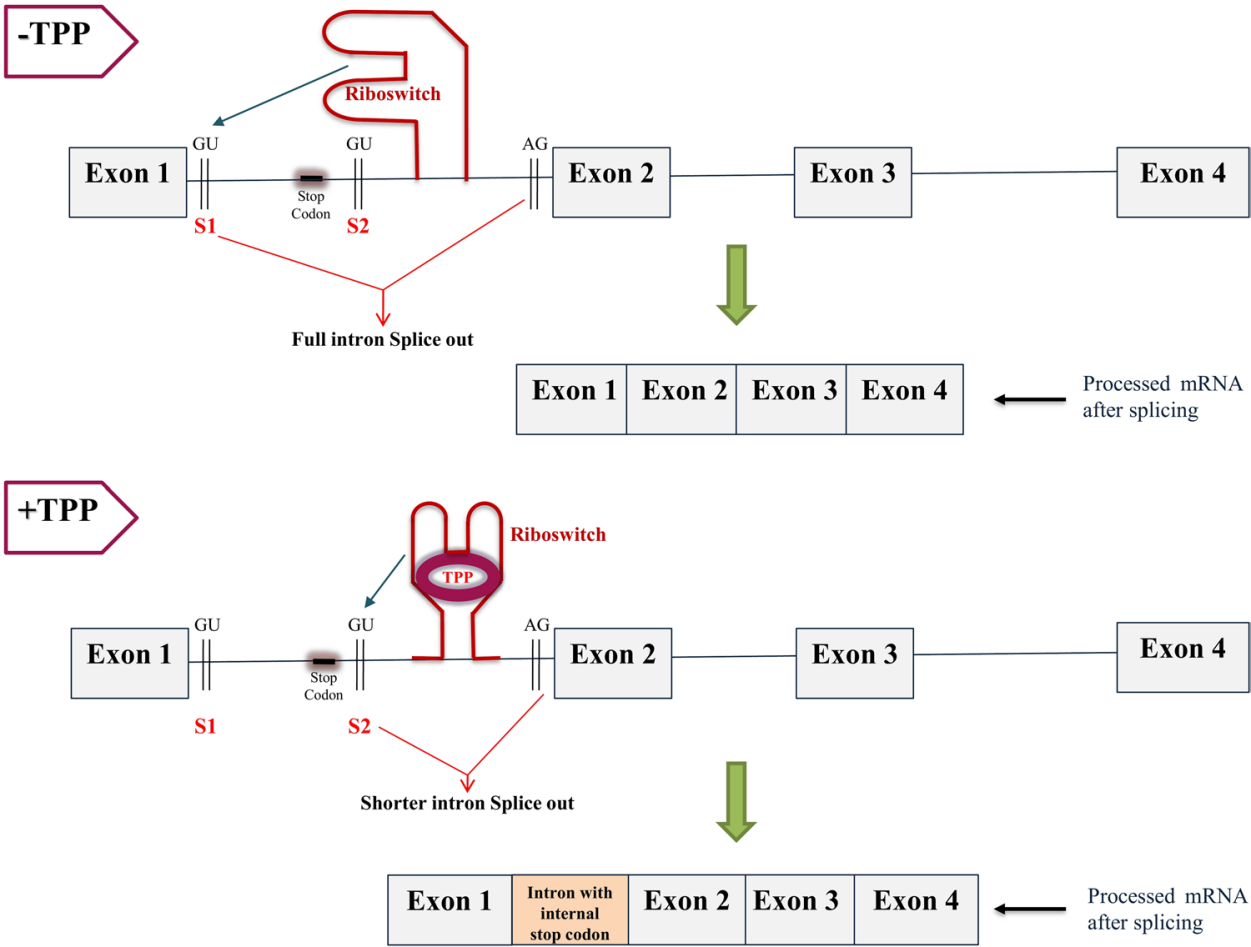
Generalized model of TPP riboswitch-based splicing (Type III) observed when the riboswitch is located in any intermediate intron of the corresponding biosynthesis/transporter genes in fungi. The top panel of the figure represents the TPP-free state of the riboswitch, which promotes the interaction with the S1 site, and splicing of the full intron containing the internal stop codon. The bottom panel of the figure represents the TPP-bound state of the riboswitch, which promotes the interaction with the S2 site and splicing of the shorter intron. The thin black line between successive exons represents the introns. The arrow pointing towards GU indicates activation of the corresponding splice site. In the TPP-bound state, the internal stop codon ensures premature translation termination resulting in a dysfunctional protein product. The thick green arrows point to the resulting processed mRNA obtained after splicing.

We have detected another mode of TPP riboswitch based splicing mechanisms (Type III) that is mechanistically similar to the Type I mode. In this type of regulation, the TPP riboswitch is present in an internal intron having length 200–400 nucleotides. This mode of regulation is facilitated by two alternative donor splice sites (S_1_ and S_2_) and a stop codon in between them in the same reading frame (see Fig. 7 and Fig. S4 of Supplementary Information). The 3′ splice site AG is found downstream of the riboswitch sequence immediately before the subsequent exon. When TPP concentration low, the riboswitch segregates the splice site S_2_, leading to splicing starting from the site S_1_ and resulting in the complete removal of the riboswitch-carrying intron, which facilitates the translation of the functional protein (see top panel of Fig. 7). But when TPP concentration is high, the expression platform of the TPP-bound aptamer changes conformation to block the splice site S_1_ and allow access to the splice site S_2_ resulting in a shorter intronic region being spliced out. The un-spliced intron portion contains an internal stop codon that interrupts the main ORF during translation (see bottom panel of Fig. 7) resulting in the synthesis of a shorter and dysfunctional peptide. Even though this mode of regulation has not been experimentally observed, it can be distinguished from Type II regulation on the basis of a shorter riboswitch-carrying intron, just two alternative donor 5′ splice sites and the presence of a stop codon upstream to the TPP aptamer. All instances of this type of regulation are listed in Supplementary Datasets S1 and S2. Examples of sequences involved in the three different modes of regulation based on alternative splicing are given in Fig. S4 of Supplementary Information, where the exons, splice sites, riboswitch aptamer, uORF and internal stop codon are highlighted.

There were 13 instances where the TPP riboswitch was found in the 5′UTR of single exonic biosynthesis or transporter genes. No 5′ splice sites (GU) or 3′ splice sites (AG) were found in the 5′ UTR of these single exonic genes. Hence the regulation mechanism is likely to be distinct from alternative splicing. Such riboswitches were found to primarily regulate the THI4 biosynthesis gene, urea transporters (see Supplementary Dataset S1). We classify these as Type IV fungal TPP riboswitches. To investigate whether there are any structural patterns that are unique for the Type IV fungal TPP riboswitches, we performed structural clustering (see methods). However, we did not find any significant cluster that can differentiate Type IV fungal TPP riboswitches from the other three types. We generated the consensus structures for Type I, II, III and IV TPP riboswitches using cmalign^45^ on the TPP covariance model from Rfam^46^ (Fig. S5 of Supplementary Information). We found that base-pair stems are highly conserved within the structures of all of these four types of TPP riboswitches. However, only the Type IV fungal TPP riboswitches have smaller internal loop compared to the other three types that are involved in splicing. We attempted to identify their regulatory mechanisms. For transcriptional regulation, the riboswitch should have two mutually exclusive RNA structures, one of which forms a transcriptional terminator, which results in premature termination and the other forms an anti-terminator that allows the coding sequence to produce a full-length mRNA. We looked for the presence of two alternative terminator-anti-terminator structures in each of these 13 cases, using the PASIFIC web-server^47^ (http://www.weizmann.ac.il/molgen/Sorek/PASIFIC/). Even though the PASIFIC web-server indicated that 4 of the 13 riboswitch sequences have such terminator-anti-terminator structures, the prediction scores were too low and fell below the threshold value of 0.5 required for reliable predictions. The low prediction score prevents us from unambiguously attributing the transcription termination mode of regulation to these riboswitches. It is possible that these 13 riboswitches regulate their corresponding genes via the translation inhibition mechanism. However, further experimental validation is needed to ascertain the precise regulatory role of these TPP riboswitches in fungi.

## Conclusions

TPP and Cobalamin riboswitches are the most common^48^ among all riboswitch classes found in prokaryotes and archaea and are ubiquitous across all prokaryotic phyla. In contrast, only a few instances of TPP riboswitches were detected experimentally in eukaryotes. Even though well-established protocols^49^ have been developed for experimental detection of riboswitches, such methods are still time-consuming and limited by the choice of model species used to search for them. Our analysis shows that computational tools can be effectively used to significantly enhance the speed and scope of discovery of these regulatory elements.

The phylogenomic distribution of TPP riboswitches in the Ascomycota phylum (Fig. 2) suggests that the origin of those regulating the THI4 gene can be traced back to the common ancestor of the following classes: Sordariomycetes, Leotiomycetes, Dothidiomycetes, Eurotiomycetes. The absence of the riboswitch in a few species belonging to these classes can be attributed either to the deletion of the THI4 gene along with the riboswitch or the presence of alternative TPP riboswitches that are sufficient to regulate TPP concentrations in these species. The same can be said for the TPP riboswitch regulating the NMT1 gene, though the absence of such a riboswitch is somewhat more common than the absence of the riboswitch regulating THI4 gene. The presence of these riboswitches in other classes of Ascomycota and Basidiomycota is more fragmented suggesting that they were most likely acquired independently in those organisms. It is also noteworthy that sequence motifs characteristic of TPP riboswitches appear to be conserved across prokaryotic and eukaryotic domains. This facilitated our discovery of new eukaryotic TPP riboswitches based on conserved patterns found in their prokaryotic counterparts. This raises the question of whether the TPP riboswitches found in eukaryotes were originally acquired from prokaryotes through horizontal riboswitch transfer along with the transfer of the regulated gene. Although our current analysis is unable to address this issue conclusively, it does reveal clues that might be useful in tackling this issue in future studies. For example, from the standpoint of the TPP riboswitch-regulated genes and TPP biosynthesis pathway, oomycetes were found to be like fungi. However, from standpoint of conservation of TPP riboswitch aptamers, the TPP riboswitch in oomycetes show higher levels of sequence similarity with Cyanobacterial TPP aptamers rather than fungal and algal TPP aptamers. This was evident from the results of both BLAST and pHMM searches using oomycetes TPP riboswitch sequences for querying or pHMM model building. The sequences returned with the highest scores, in either case, were TPP riboswitches from Cyanobacteria.

To further investigate whether there are any structural patterns that are unique to fungal and oomycetes TPP riboswitches, we performed structural clustering (see Methods section) of TPP riboswitches. Multiple runs of increased clustering thresholds, between 30 and 50, generated unspecific clusters with unstructured or single motif conservation. Moreover, no cluster had a significant group of sequences belonging to the same group where grouping was done based on the mode of regulation, intronic location, and gene function. These observations further indicate that fungal and oomycetes TPP riboswitches are similar at both the sequence and structural levels with the bacterial TPP riboswitches and lends support to the hypothesis that fungal and oomycetes TPP riboswitches share a common origin with their bacterial counterparts.

The location of riboswitches and their genomic context aids in the identification of TPP transporters and reveals the mechanism of regulation. We found TPP riboswitches in the 5′ UTR, 3′ UTR, as well as the intronic regions. Additional identification of uORF, number and location of splice sites, the length, and in some cases sequence motifs present in the riboswitch-carrying intron, enabled us to draw conclusions about the mechanism by which each riboswitch operates. Our analysis reveals that TPP riboswitches in fungi and oomycetes are not just involved in alternative splicing but can also potentially regulate single exons via alternative mechanisms such as translation inhibition. However, the precise mechanism of regulation of this new class (Type IV) of fungal TPP riboswitches needs to be experimentally ascertained.

The results presented in this paper underscore the usefulness of a comparative genomics and phylogenomic approach to discovery, annotation and functional classification of regulatory RNA. At the same time, we believe it will facilitate a more rational and focused approach to experimental validation and closer examination of riboswitches in species of biotechnological importance.

## Methods

### Collection of sequence data

A total 192 complete fungal genome sequences and 17 complete oomycetes genomes were retrieved from RefSeq database^50^, JGI MycoCosm web portal^18^, and FungiDB^51^. The genomes were categorized into different classes based on their taxonomy. For the cases of multiple strains belonging to the same species, we found that all the strains from a species have the same pattern for the presence/absence of TPP riboswitches. Therefore, to avoid redundancy in our analysis, we considered only one representative strain for a species. Shell scripts were used to extract and categorize the genomic data. BEDTools^52^ were used to compare and map the sets of genomic features. NCBI ORFfinder was used to detect the uORF from the UTR sequences.

### Identification of TPP riboswitches

The aptamer domain of TPP riboswitches shares a high of level sequence conservation across distantly related organisms. Previously, we had exploited this characteristic to accurately detect riboswitches using pHMM’s^26,27^. Hence, TPP riboswitch specific pHMM was downloaded from Riboswitch Scanner^27^ and used to search against all the complete fungal and oomycetes genomes. For further validation of TPP riboswitches detected by pHMM, we constructed the TPP riboswitch specific Covariance Model (CM) using the Infernal software package^45^ and searched against the pHMM predicted sequences. The CM also validated all the TPP riboswitches detected by our pHMM model. We found 291 TPP riboswitches across all fungal species (not counting those detected in different strains of the same species) and 37 in oomycetes. To consider potential false negatives during the process of TPP riboswitch detection using pHMM, we took all the fungal species where no TPP riboswitch was detected by pHMM, and additionally scanned them by using both CMs and RiboSW^53^. Since we did not find any new TPP riboswitch, we were able to confirm the absence of TPP riboswitch in those organisms.

### Structure-based energy minimization method for validations of TPP riboswitches

To further verify the riboswitches found to regulate the hypothetical proteins and predicted transporter genes, we used a structure-based riboswitch detection method^32,33^ that incorporates energy minimization technique to predict the folding patterns which generate the optimal and sub-optimal secondary structures for the potential riboswitch candidates. These folding predictions obtained on the basis of energy minimization can be computed with UNAFold^30^ or the Vienna RNA package^31^ and can be compared to the reference riboswitch structure derived by comparative analysis or x-ray crystallography/NMR experiment. We chose to work with UNAFold^30^ for folding predictions and to compare those folding patterns to the structure of the *Neurospora* TPP aptamer that is depicted in Fig. 1 of *Sudarsan et al*.^6^, and the crystal structure of the eukaryotic TPP aptamer described in *Thore et al*.^35^. VARNA^54^ was used to generate the secondary structure of riboswitches.

### Structural clustering of TPP aptamers

Structural clustering of the TPP riboswitches was performed with multiple cluster threshold based on the Infernal^45^ cmscore. The tools selected were RNAscClust^55^ and its predecessor GraphClust^56^. GraphClust^56^ input was the entire set of sequences while RNAscClust^55^ received multiple sequence alignment for each of the three groups (bacteria, fungi, and oomycetes). In this analysis, the lowest selected threshold was set to 20 which is half of the TPP family co-variance model cutoff score according to Rfam^46^. Using this score the tool generated a single cluster matching the structure for the TPP riboswitch family. Multiple runs were performed by gradually increasing the thresholds to check if the TPP aptamers can be distinguished on the basis of the group, intronic location, and function of the regulated gene. Additional manual alignment based on the mode of regulation, intronic location, and gene product was attempted using Infernal’s^46^ cmalign programs. While the alignment shows high preservation of general motifs, we observe changes in loop sizes and the introduction of small interior loops and bulges in the stems.

### Annotations of transporter genes

TPP riboswitches are found to regulate some genes that have no functional annotations and are labeled as hypothetical proteins. Our systematic comparative genomics approach enabled us to annotate these genes as TPP transporters. We first carried out the BLASTP^34^ search of all those hypothetical proteins versus all the transporter proteins in the TCDB (Transporter Classification Database)^57^ to identify the homologs to known and predicted transporters in the TCDB. The parameters set to consider homologous genes were as follows: E-value ≤10^−5^, similarity ≥50%, and the sequence coverage ≥30%. We have functionally annotated only those hypothetical proteins as transporter genes for which their BLASTP search yielded a homologous gene with known transporter function in the TCDB. Next, the Pfam database^58^ (http://pfam.xfam.org/) was searched to detect the conserved structural domains of the transporters and TMHMM^59^ (http://www.cbs.dtu.dk/services/TMHMM/) search was used to analyze many transmembrane domains in the transporter proteins. Only those hypothetical proteins that have conserved structure domains associated with transporters, as well as transmembrane domains, are labeled as putative TPP transporters.

### Selection and validations of the phylogenetic marker genes for fungal tree of life

For phylogenetic tree construction, four proteins were selected to build a highly resolved fungal tree of life. Only those single copy proteins that were found in all classes of fungi were used for this purpose. A first step in the selection process was to identify genes present in single copy in all fungal genomes. This was done by performing a BLASTP search, from a seed species, against all other fungal genomes, and selecting those proteins with a single hit (E-value cut-off 10^−5^ and coverage > 50%) in every other genome. (The seed species were randomly selected and more than one seed can be used to increase the number of detectable single-copy proteins). Further, OrthoMCL^60^ search was performed to confirm the single-copy ortholog proteins detected by BLASTP searches that are present across all the fungal classes. Only four single-copy proteins were found to be common across all the fungal genomes used in our dataset.

These phylogenetic marker genes (TSR1: OG5_128208, MCM2: OG5_127764, SecY: OG5_127102, YchF: OG5_126949) selected in our study were used to construct the individual Ascomycota and Basidiomycota tree. A systematic analysis^61^ was carried out to validate the ability of the selected marker gene sets to resolve the fungal tree. All the fungal genomes were randomly split into two sets, of sixty species each, containing species from all the fungal phyla and its representative classes. Phylogenetic trees constructed for each set were well resolved with high bootstrap values and produced distinct clusters of species belonging to the different fungal phyla (see Fig. S6 of Supplementary Information). Our results show that the four selected gene markers, used in combination, have a strong potential to reconstruct accurate phylogenies of fungal species and that they will be valuable in reconstructing a complete and well-resolved fungal tree even as the number of sequenced fungal genomes keep increasing over time.

### Construction and visualization of phylogenetic tree

Phylogenetic trees were constructed for Ascomycota and Basidiomycota classes. The protein sequences corresponding to each phylogenetic marker were extracted from all the species and aligned using MUSCLE^62^ and the poorly aligned regions with more than 20% gaps were trimmed using trimAl^63^. The aligned sequences were concatenated to produce a super-gene alignment that was then used to build a phylogenetic tree using neighbor-joining (NJ) as well as maximum likelihood (ML) methods. The NJ and ML trees were generated with the MEGA 7 package^64^. The evolutionary distances were computed using the JTT matrix-based method and are in the units of the number of amino acid substitutions per site. Both trees were generated for 100 bootstrap replicates. The two trees were found to be consistent with one another except for some rearrangements in some of the late branches and differences in some bootstrap values that do not affect our conclusions. The mapping of riboswitch distribution on the phylogenetic tree was visualized using iTOL v3^65^. Filled shapes (circle for biosynthesis and square for transporter genes) indicate the presence of a riboswitch upstream to the corresponding gene. Bootstrap fractions greater than 0.5 (corresponding to a bootstrap value of 50%) are denoted near the branch points of the phylogenetic trees.

### Data availability

Data supporting our findings are provided in the supplementary dataset files.

## Electronic supplementary material

Supplementary Information

S1

S2
