# Supplementary material for: Phylogenomic and comparative analysis of the distribution and regulatory patterns of TPP riboswitches in fungi
Source: Sci Rep. 2018 Apr 3;8:5563. doi: 10.1038/s41598-018-23900-7 (PMC5882874; doi:10.1038/s41598-018-23900-7)
Supplement: Supplementary file 1 — Supplementary Information [file 41598_2018_23900_MOESM1_ESM.pdf]

Supplementary Information

Phylogenomic and comparative analysis of the distribution and regulatory patterns of TPP riboswitches in fungi

Sumit Mukherjee[1], Matan Drory Retwitzer[2], Danny Barash[2] and Supratim Sengupta[1*]

1 Department of Physical Sciences, Indian Institute of Science Education and Research Kolkata, Mohanpur-741246, India

2 Department of Computer Science, Ben-Gurion University, Beer-Sheva, 84105, Israel

Figure S3

Figure S3: RNAfold generated secondary structures of the TPP aptamers found to regulate the THI4, NMT1 and urea transporter genes in *Neurospora crassa* are depicted. The corresponding multiple sequence alignment (MSA) of the intronic locations of THI4, NMT1 and urea transporter genes where the TPP riboswitch is located are also shown. Conserved structural features of the TPP aptamers are highlighted in the multiple sequence alignment.

<u>Figure S3-1:</u> Structure of TPP aptamer involved in regulation of THI4 gene in *Neurospora crassa*

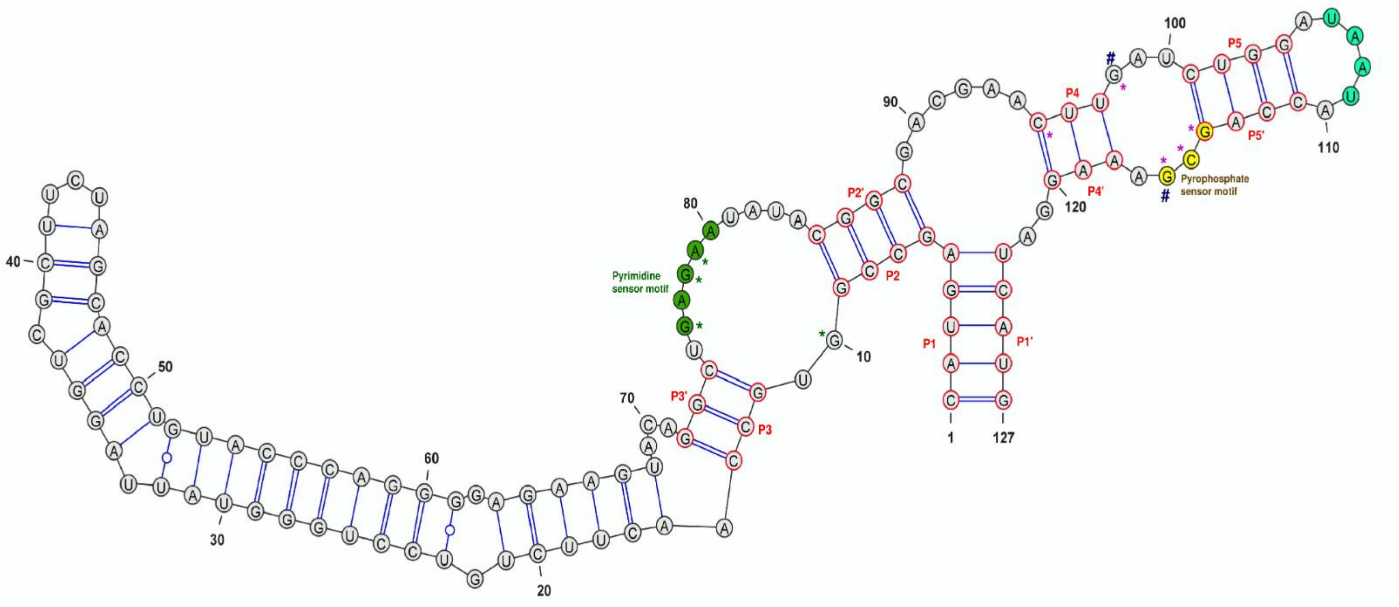

- RNAfold was used to predict the minimum free energy structures and base pair probabilities. VARNA was used to draw the structure. The bases making up the stems (P1 to P5) which are conserved across phylogeny are highlighted by red circles. Bases which are responsible for pyrimidine recognitions are marked with green stars and bases responsible for pyrophosphate recognitions are marked with purple stars. The guanine bases at position 98 and 116 (highlighted with navy-blue #) form a non-canonical G-G base-pair upon TPP binding. The conserved GAGAA motif which recognizes the TPP aromatic ring are highlighted in dark green. The conserved GCG motif that interacts with the pyrophosphate moiety are highlighted in yellow. The conserved UAAU motif that is responsible of the complete closing of the two halves of the aptamer after initial binding of the aromatic ring to the GAGAA motif are highlighted in cyan.

Figure S3-2: MSA of a fungal intronic regions of the THI4 gene where a TPP aptamer is found. *Neurospora crassa* is used as the reference species. Conserved structural features that are consistent with phylogeny are highlighted with different colours on the alignment.

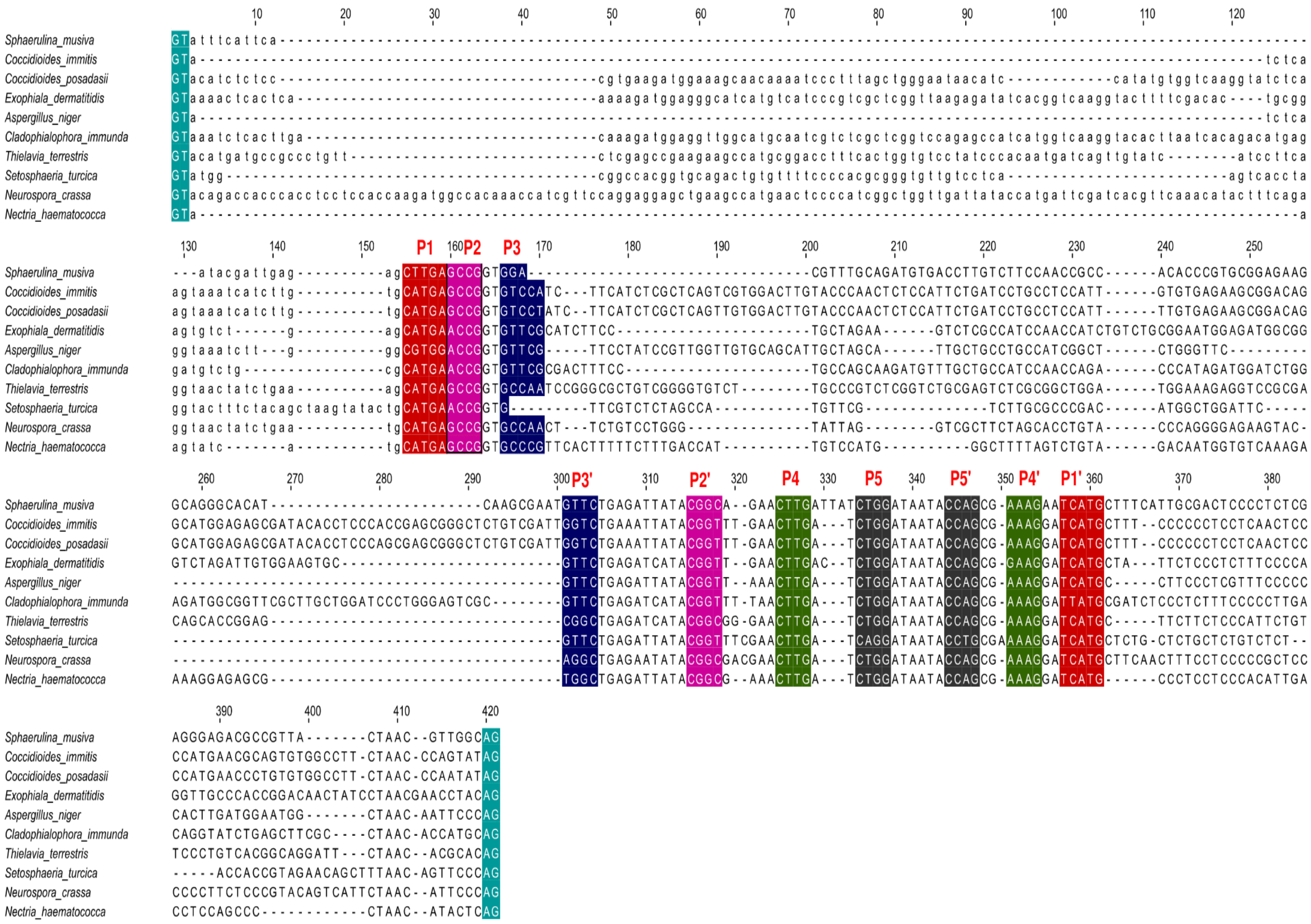

Figure S3-3: Structure of TPP aptamer involved in regulation of NMT1 gene in *Neurospora crassa*

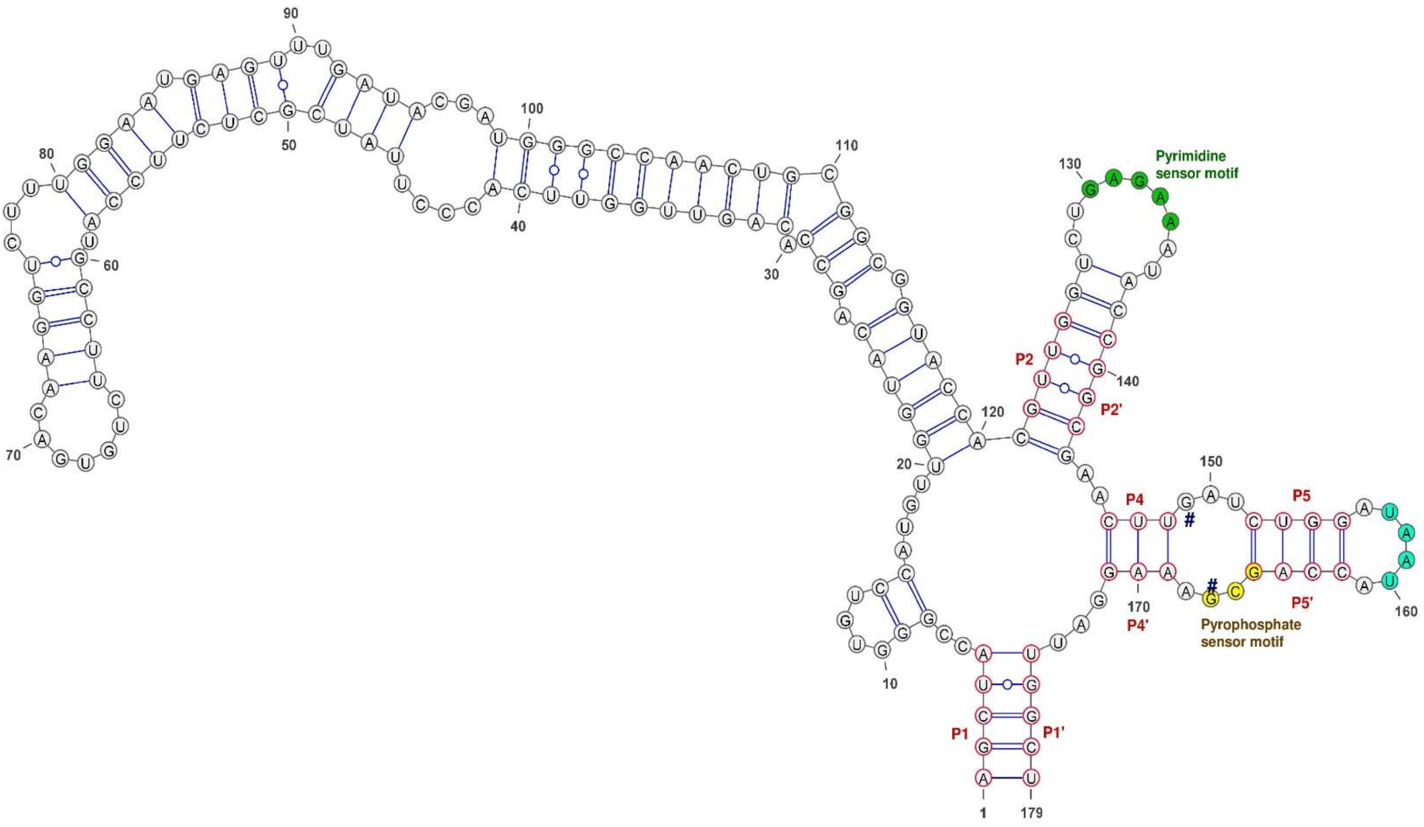

- RNAfold was used to predict the minimum free energy structures and base pair probabilities. VARNA was used to draw the structure. The bases making up the stems (P1 to P5) which are conserved across phylogeny are highlighted by red circles. The conserved GAGAA motif which recognizes the TPP aromatic ring are highlighted in dark green. The conserved GCG motif that interacts with the pyrophosphate moiety are highlighted in yellow. The conserved UAAU motif that is responsible of the complete closing of the two halves of the aptamer after initial binding of the aromatic ring to the GAGAA motif are highlighted in cyan. The guanine bases at position 149 and 167 (highlighted with navy-blue #) form a non-canonical G-G base-pair upon TPP binding

Figure S3-4: MSA of a fungal intronic regions of the NMT1 gene where a TPP aptamer is found. *Neurospora crassa* is used as the reference species. Conserved structural features that are consistent with phylogeny are highlighted with different colours on the alignment.

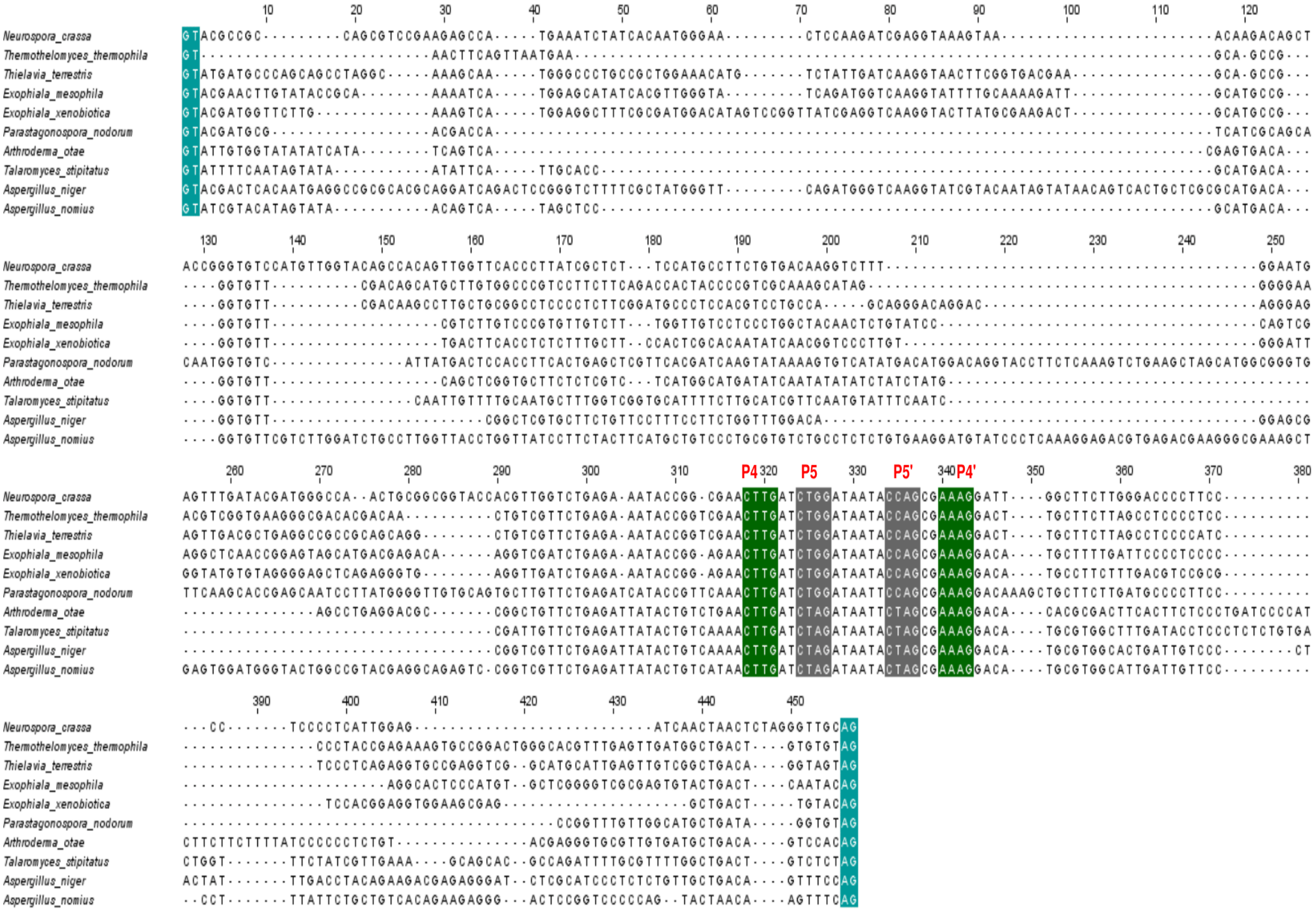

<u>Figure S3-5:</u> Structure of TPP aptamer involved in regulation of transporter gene belonging to the urea transporter family in *Neurospora crassa*

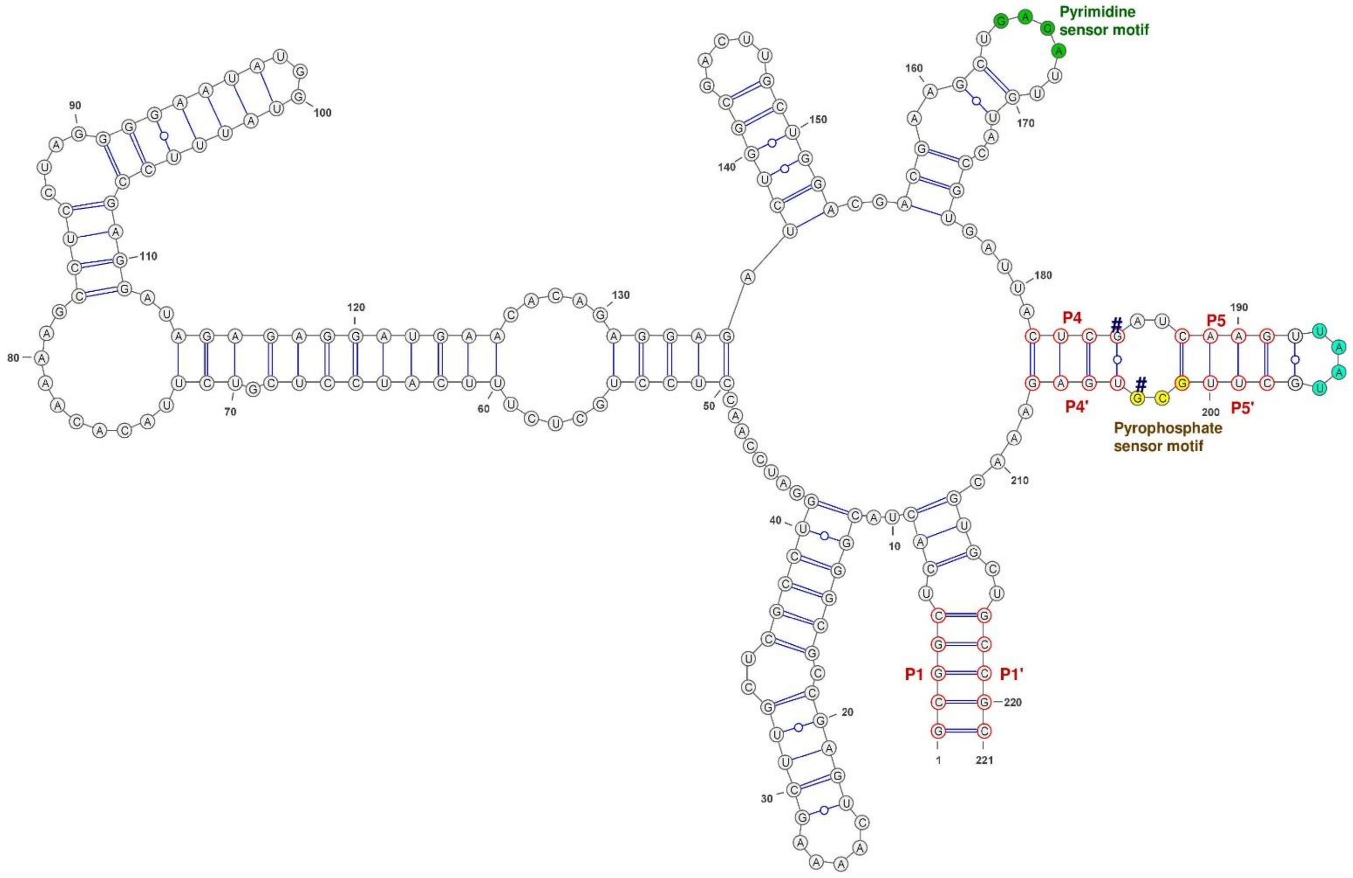

- RNAfold was used to predict the minimum free energy structures and base pair probabilities. VARNA was used to draw the structure. The bases making up the stems (P1 to P5) which are conserved across phylogeny are highlighted by red circles. The conserved GAGAA motif which recognizes the TPP aromatic ring are highlighted in dark green. The conserved GCG motif that interacts with the pyrophosphate moiety are highlighted in yellow. The conserved UAAU motif that is responsible of the complete closing of the two halves of the aptamer after initial binding of the aromatic ring to the GAGAA motif are highlighted in cyan. The guanine bases at position 185 and 203 (highlighted with navy-blue #) form a non-canonical G-G base-pair upon TPP binding

Figure S3-6: MSA of a fungal intronic regions of the transporter gene belonging to the urea transporter family where a TPP aptamer is found. *Neurospora crassa* is used as the reference species. Conserved structural features that are consistent with phylogeny are highlighted with different colours on the alignment.

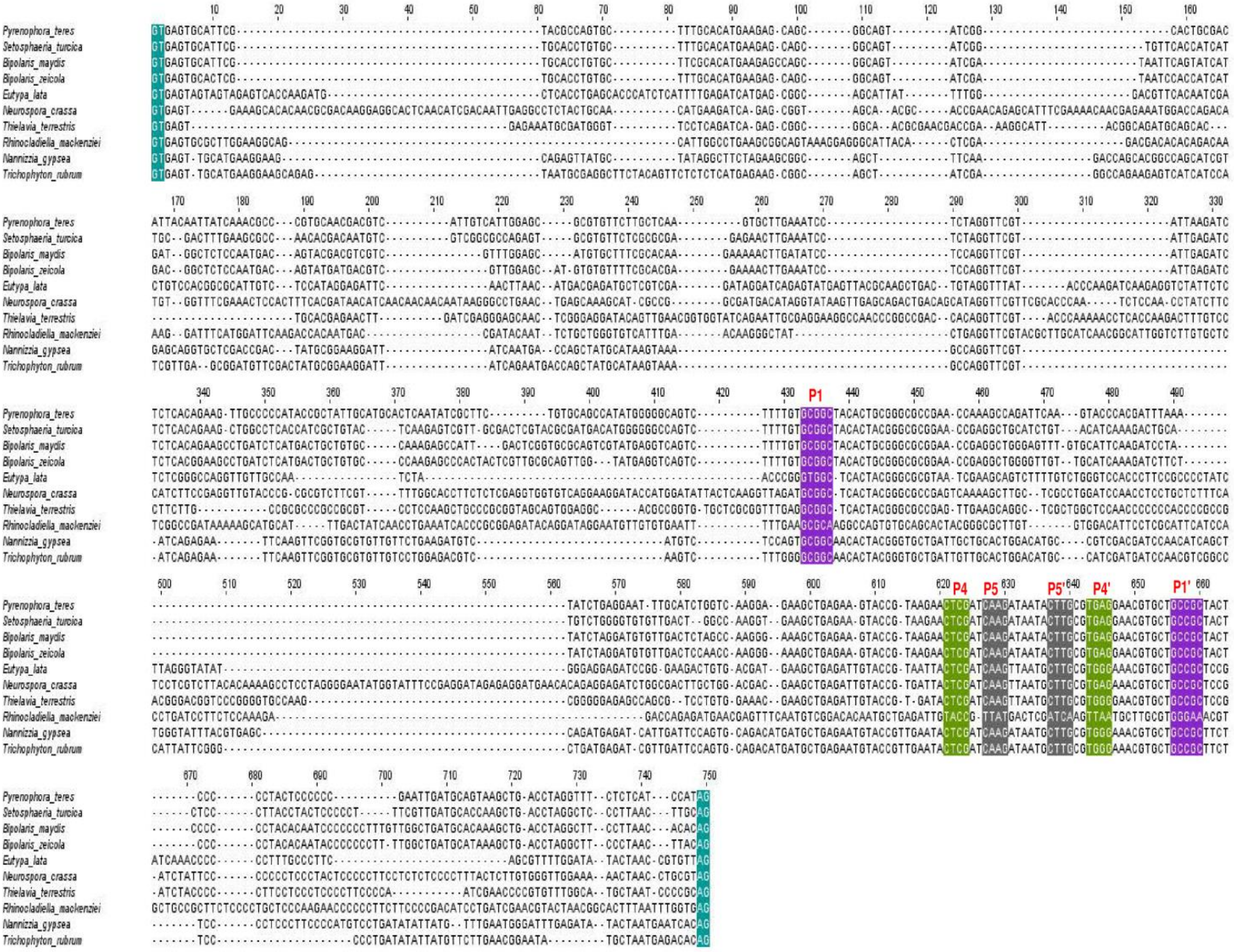

Figure S4

Figure S4: Examples of sequences involved in three distinct types of TPP riboswitch- based splicing mechanisms. The riboswitch sequence, splice sites, uORF (where applicable), internal stop codon (where present) and exons have been highlighted in each of the examples.

TPP riboswitch mediated Type I Splicing

Figure S4-1 (TPP riboswitches in 5' UTR of NMT1 gene)

```
>NW_001517110.1:708600-709018 Aspergillus_clavatus NMT1
GTCAGGATGGCCCTCTGGGCCGATTTCGCTAGGGAAATCATGCAGATCAAGGTATCGTACAACGTATAAT
AATGGTCACTGCTCGCGCATGACAGGTGTTCGGTCCGGGGGTTTCTAGCTGGATAGCTACCTATAGGGGT
TTCCCGTTTGGCGAACTCCAGCTCATCCAGCTAGAGAACCCGGCCCGTTCTGAGATTATACTGTCATAAC
TTGATCTAGATAATACTAGCGAAAGGACATGCGTCGCACTGATTGTCTCACCCTTAATGGGTTCCCCCCC
CACCCCTCTCTGCTTGCTGACGAGTAGCTCCAGACAATTGCAAGAACTGCCGTTACGGAAGCCTGTCTAT
CAAGATGTCGACTGATAAGATCACCTTCCTGACCAACTGGTACGGCTTCTTGCGGTTGTTGATGAAAGAC
GGGAACTGTGCTAAATTACTTTGTTTTTTTCTTTCTTTTCTTGTTATAGGCACGCAACCCCCTACCATGC
CCCCCTGTACCTTGCCCACAGCAAGGGTTACTTCAAGGATGAGGGCCTGAAGGTCGCTATCCTTGAGCCC
AATGACCCCTCCGATGTGACCGAGATCGTCGGTAGCGGCAAGGTTGACATGGGCTTCAAGGCTATGATCC
ATACCCTAGCTGTATGTTCCTGTGGTTCGGAAATGCTTGTATGACTCGTGTACTGACTGGCTCCCAGGCC
AAAGCCCGCAACTTCCCCGTCACCTCGATCGGCTCGCTTCTTGACGAGCCCTTCACCGGTGTCATCTACC
TCAAGGACAGCGGGATCACCGAGGACTTCCGCTCTCTCAAGGGCAAGAAGATCGGATATGTTGGAGAGTT
CGGCAAGGTTGGTTCCAACCACTCTGGGGTCCAATTGACATGTGCCGACTGACACACGGAGTAGATCCAA
ATCGACGAGCTTACCAAGCACTACGGCATGACCGCCGACGACTACACCGCCGTCCGCTGCGGCATGAACG
TGACCAAGGCCATCATCCGCGGCGACATTGACGCGGGCATCGGCCTCGAGAACGTGCAGATGGTCGAGCT
GGCCGAGTGGCTGGCGTCGCAGAACCGTCCCCGCGATGACGTCAAGATGCTGCGCATCGACCAGCTCGCA
GAGCTGGGCTGCTGCTGCTTCTGCTCGATCCTCTACATCGCCAACGACGCCTTCCTGGCCGCCAACCCAG
AGAAGGTGCACAAGTTCATGCGCGCCGTCAAGCGCGCCACCGACTTCGTGCTCGCCGAGCCCGCCAAGGC
CTACGAGGAATACATCGACGTCAAGCCCATCATGAACACCCCCGTCAACCGCAAGATCTTCGAGCGCTCC
TTCGCCTACTTCAGCCGCGACCTCAAGAACGTCCAGCGCGACTGGACAAAGGTCACCAACTACGGCAAGC
GCCTCGAGATCCTCGACGCCGACTTCCAGCCCAACTACACCAACGAGTTCCTCTCCTGGACCCTGGAAGC
AGACTCCACCGACCCGCTCGGCGACCAGAAGCGCATGGCCCAGCTGCAGCAGGAGGTCGCGCTCAATGGT
GGCTTCAAGCGCCTGCAAGTCTCCGCTACGGCTTAG
```

Figure S4-2 (TPP riboswitches in 5' UTR of THI4 gene)

```
>NC_018213.1:294450-294768 Mycosphaerella_graminicola THI4
GTGACTTCCACCCTGGACGATGATGAATGTTGCGGTCCATCCTTCCCGATGAGATACCTCTCTCCACATC
ACCGTCCTGGAGAAGTCGCAGCACCACCACGAGTCGTTCTGAGATTATACGGCAGAACTTGATCTTGGTT
AATACCAGCGAAAGAATCATGCTCAACATCCCCAATCTCTCACGAGGCAAATACTGATCAACCTTAGTTC
ACCAACGACAACAACCATAAGCAACCATGTCTCCACCAGCTGCCATGTTCCAGGAGCCCGCCTCCATCCC
AGTGATGCCCACCAAAGGCACCAAGCCGACAGCCACCATCGAACAGATGGCCAACAACTGGTCCTCGTTC
AAGTTCGCTCCCATCCGCGAGTCTCAGGTGTCCCGGGCCATGACTCGCCGCTACTTCAGCGACCTGGACA
CCTACGCCGAGTCCGACATTGTCATCGTCGGTGCTGGAAGCTGCGGTCTCTCCGCCGCCTACTGTCTCGC
CAAAGCCCGACCAGACCTCAAGATCGCCATCATCGAAGCCGGCGTGGCTCCGGGCGGAGGTGCCTGGTTG
GGAGGCCAGCTGTTCAGCGCCATGGTCATGAGGAAGCCTGCCGATGCTTTCTTGCGAGAGATCGGTGTAC
CGTACGAGGACGAAGGTCCCGACTCGAACTTTGTGGTCGTCAAGCACGCTGCTCTGTTCACCAGCACTGT
GCTATCCAAAGTGCTGCAATTCGACAACGTCAAGCTCTTCAACGCCACCACGGTCGAGGACCTCATCACC
CGTGCCGATGACCAGGGAAAGCTTCGCATCGCTGGCGTGGTCACCAACTGGACTCTCGTCAGCATGCACC
ACGGTAAGACCCATCCACCACTCTCATCCTCCACCACCAGACTAACCTCCCCTCAAGACGACCAATCCTG
CATGGACCCCAACACCATCAACGCCCCCATCATCATCTCCACCACGGGCCACGACGGTCCTTTCGGCGCC
```

TTCTGCGCCAAACGCCTCGTCGCCACCGGCATGCTGTCCCAACTCGGCGGCATGCGCGGCTTGGACATGA
ACACCGCCGAAGACGCCATTGTCAAGAACACCCGTGAGGTCGCTCCGGGTCTCATCATTGGCGGCATGGA
GTTGAGCGAGGTCGATGGCGCGAACCGTATGGGCCCGACGTTTGGCGCTATGGCGTTGAGTGGTGTCAAG
GCTGCGGAGGAGTGCTTGAAGGTGTACGAGGAGAGGAAGAAGCAGAATGCTTTTTAA

Figure S4-3 (TPP riboswitches in 5’ UTR of transporter gene)

>NW_014024910.1:53800-54051 Bipolaris_maydis nucleoside transporter
GTACGGCATTGCAGCCATGATGCAGCCAAGTAGCAGATTGTCGCCCGATCAAGGTAACCTCAGGTAA
CACAATGCATGAGACCG**GTGT**TCGCTCGCCATCCATTTCCTTTGTGGATGGCAGCGATCTGAGATAT
ACGGTCTGAACTTGATCAGGTTAAAACCTGCGAAAGGACTCATGCTTTGCTCCCTTCACAAATTGGG
GGCTGCTGCTAATGGCTCT**AG**GTCGGTGCCATGGACTCTATCGCGAAGCCGCACGATGAGAAAACGT
CAGATGCGCCGGGCGCAAGTAGTTCACCATTGACTCACGATATCGAGCGCCAGGATGATGTGCGCTA
TGCAAAATGGCAAAAGACTTGGAAGACATTCGAGAGCCAGCTTGTAGCATACAACCTCGAGGCCCGC
GGCATTCAGCGCGTCGAGCCCCACGAACGTCATGATTTGCGCCTTTTGGGGTATTCGCAGGTCGCAA
TCATGTGGTTTAGTGTCAACTTGGCAGCGAACAACATCACACTGGGCATGCTTGGCCCTGCCGTTTT
CGCCCTCGGCTTTACAGACGCCTGTTTACTGAGCGTCTTCGGTGCACTGGTTGGTTGCCTCGTAGTC
GCCTATGTCGCCACATTTGGTCCGAAGAGCGGTAATCGCACCATGATCTTCTCGCGGTACATTACGG
GTTGGTGGCCATCCAAGATCATTGTGTTACTCAATATCATCGTACTACTCGGATATGGCATGATCGA
CTGTGTAGTAGCAGGCCAAATCCTCTCTGCTGTCTCTACAAATTCCATGTCTGTCGTGGTTGGCATC
ATTATCGTGGCCGTCATTGCTTGGGCAATTACCACATTCGGATACCAGATATTTCACTATTACGAAC
GTTGGGCATGGCTGCCGCAGCTCGTAGTACTCTGCATATTGGCGGGCGTCGCAGGCCCGCGATTTGA
CATATCTTCCAAATCTTATGGCGACGAAAATCCAAATACCATCATCGGGAATCGCATCAGCTTTTTT
GGCCTTACGCTTGCAGCAGCGATCACGTACGGTGGTGGTGCAGCCGACTACTTCGTCTACTATCCTG
AACATGCATCGTCTCTAAGCATCTTCGGCATGACCCTGATTGGCCTAATGTGTAGTTTCACATTCGC
TTTCGTCCTTGGTATTGGGCTGGCATCCGGCATGTCGAACAATGCAGACTGGGAAGCAGCGTACGGC
GTATCGCAAGGTGCGCTTATTGTGGAGGGATACAAGCCTCTCGGTGCATTTGGCTCTGTTTGTGGCG
TCATTGTCGCTTTGGGACTTGTCGCCAACCTGATTGTACCAACATACTCTTCTGGAATTGATGCACA
GATCCTCGGTCGTTATGCCGCTGCCATACCAAGAGTGATCTGGAATACCGTCGGCGTCATCATCTTC
ACAGTCTGCGCCCTGGCAGGGCGTGCGCACCTTGCGGAAATCTTTACCAATTTCCTAGCCCTCATGG
GCTATTGGGTGTCTATCTGGATTGCCATTATCCTAGAAGAGCATTTGATCTTCCGCCGCAAGACAGG
CTTCAACTGGGAAGTATGGAACCAGAAGAAGAAGCTACCACTGGGCATCGCAGCATTGGCTGCCTTT
GTCATCGGTTGGATTGGCGCCATCATGTGCATGGCACAAGTCTGGTACATTGGGCCTATCGCCAAAC
AAGTTGGAACTCACGGCGCTGAC**GT**ATGTGAATTGCATGTGCAAGCTTTGGTAACTCATACTAACAC
CTTCT**AG**ATGGGTAACTTTGTCGGTTTCGCATGGGCTTCGGTCGTGTATCCGCCGCTCAGGATGTGG
GAGCTTAAGCGATTCGGCAGATGA

Description:

In the sequences, the region highlighted with grey indicates the exons, region highlighted with yellow denotes the TPP riboswitch and region highlighted with pink indicates the uORF. Splice sites GT and AG are highlighted in red and viable alternative 5’ splice sites GT highlighted in cyan..

TPP riboswitch mediated Type II Splicing

Figure S4-4 (TPP riboswitches in long intron of urea transporter gene)

>NC_026501.1:c1802672-1799279 Neurospora_crassa Urea Transporter
ATGAGAATTTCACTTTGCTTCTAAGAGGAAACGAAGGAGGACAATGACGGAAACTGTCTAACCTCAATAA
TCACCAGAACCGTCATCCGTCAATTGAATTTGTGTGGACTTTTATTCGCTGCTTTCTCGCTTACTTTTCG
ACTGTTCTTGCTTCAGTTCTTATCGAGTCCATTCTGGGGCAAAGCGCCAATCGTTTCGACGGAGAAGCAC
CGAAAGCCCCACCATTACGCCCGCTCGCTTAAAAGAAAAGAGAACCTCGACAGTTCAACAACTTTTTCCG
TCGCAACATCGTCCTCAGCAACCACTGGAGTCTGTTTGTATTCCAAGCAAAATCGTACCAGAACAATGGG
TCAACCATCCTCCGAGGCGTCGAACGCCATCATCTATACTACATTGGGAGTTTTCCTGTGAGATGACAAA
CACCAAACCCCGTTTTAAGCCAGGTCGGTACGGGGGAGCGTTCATACTGACTTATCGCCGCAGGATCATG
GGAACCGGTGTTGCCTGGACGATGAGAAACCAATCCAAGGGGGATTTCCTCGCCGGAAACAGGACTCAGA
CGGGTTAGAAGACCTTCCTGCTAGGCTCAGACAAGAGAGACTGCAATCCCCTGTTCTGTCGAGCCATCAC
CTACTCACACAGCCTTCCGTTGCCACTTACAATACCTTGAGCCGCAACTAACATCCCACTCTCTTCTAGC
TATTCCGCTCGCTTTGAACTTCATCGCTTCCGGTGAGTGAAAGCACACAACGCGACAAGGAGGCACTCAA
CATCGACAATTGAGGCCTCTACTGCAACATGAAGATCAGAGCGGTAGCAACGCACCGAACAGAGCATTTC
GAAAACAACGAGAAATGGACCAGACATGTGGTTTCGAAACTCCACTTTCACGATAACATCAACAACAACA
ATAAGGGCCTGAACTGAGCAAAGCATCGCCGGCGATGACATAGGTATAAGTTGAGCAGACTGACAGCATA
GGTTCGTTCGCACCCAATCTCCAACCTATCTTCCATCTTCCGAGGTTGTACCCGCGCGTCTTCGTTTTGG
CACCTTCTCTCGAGGTGGTGTCAGGAAGGATACCATGGATATTACTCAAGGTTAGATGAGAGGTTGCGGC
TCACTACGGGCGCCGAGTCAAAAGCTTGCTCGCCTGGATCCAACCTCCTGCTCTTTCATCCTCGTCTTAC
ACAAAAGCCTCCTAGGGGAATATGGTATTTCCGAGGATAGAGAGGATGAACACAGAGGAGATCTGGCGAC
TTGCTGGACGACGAAGCTGAGATTGTACCGTGATTACTCGATCAAGTTAATGCTTGCGTGAGAAACGTGC
TGCCGCTCCGATCTATTCCCCCCCTCCCTACTCCCCCTTCCTCTCTCCCCTTTACTCTTGTGGGTTGGAA
AAACTAACCTGCGTAGCTCTCGGTTCTGGAATCCTTTTCACTTATCCAGAAATCGCCACGTTGGCTGGTC
TTCAGGGCGTCCTTGTCTATGCTTTGGCTTCCGCACTCCCGCTGTTCGTCTTCGCCTTACTGGGCCCTAT
CATCAGACGCAAATGCCCTGAGGGTTTCGTCCTCACGGAGTGGACCAGACAGCGCTATGGAACCATCGCG
GCCTTGTACCTGAGCTTTGTGACGCTGGTTACGCTTTTCCTGTACATGGTTGCTGAACTGTCCGGTATTG
GTCAGGTTGTTGAAGTCTTGACTGGTCTTAACGGATTGCCGGTCCTCATCGTCGAGTGTGTAATCACGAC
CATCTATACCTGTATGTGTCTCTTCTTTGAACCCGTCGAACCCGTCAAATGATTGAATAAGTGCTGACGT
GGGTGCAACCTGCAGCATTGGGCGGGTTTCGAATCTCTTTCATTACAGACAACATTCAGGGAGCGATGGT
GATCGGACTCCTCGTCGTCGCATCCATTGCTATCGGCGTCGAGACCAAGATCGATACCAGCCTGATCGAG
CCCTCTGGTCTTTTGAAGGATAGTCTCTTGGGCTGGCAGCTTCTCTACATCCTCCCGGTTGCCATTTTGA
CGAACGACTTCTTCTTGGTAAAGTCTTCCCTTGCCGAACAGGAACCGGCCCATTGTCACTTGACTAACAC
CCAAGCAGTCCAGCTTCTGGCTCCGCACCTTTGCCTCCAAGACCGACCGTGATCTCTGGATCGGCATCAC
CCTTGCTGCCCTTTTTATCCTCATTATTATCACCATGATAGGCTGCACCGGACTTATCGCCGCCTGGTCC
GGCGTCTGGCCCGGCTCGGACCCGGAAAACCCCCTCCCGGGTTCCGTTGCCTTCTTTGGCCTGCTTGAGA
ATCTTCCTGCCTGGGTCGTCGGCTTCGTCCTCGTCATGTCTGTCACCCTCAGCACCGCCGCCTTTGATTC
GCTCCAGTCCGCCATGGTCTCGTCCGCCAGCAACGATTTGTTCCGCAACAAGCTCAGCGTGTGGTGGATC
CGCGTCGCCGTAGTTTTGATCATCATCCCCATCGTGGTCCTTGCCATCAAGGCGCCCTCGATTCTGCAGA
TCTACCTGATTTCCGATCTCGTCTCTGCCGCCACCATTCCCGTGCTGATCGTTGGCTTGTCCGACCGGTG
CTACTGGTGGCGCGGCTTTGAGGTGGTCGTCGGCGGGTTGGGCGGTATCTTCACCGTCTTCATCTTCGGC
GCGATCTACTACAACGACGCATACAAGGGCGCTCAGCTGATACTACTCGAAGACGGCATCTATCAGGAGG
GCTGGGCCGCGTTCGGTGCCTTCGTGGCTGCCCCTGTCGGCGGTCTTTTGTGGGGCTTTGGAGCCCTTGC
GCTCAGGCTGGCGGTGCAATGGGTCCAGGCCAAGAGAAAGGGTGTGAGGTTCGACGCCCTTGACCGTCCA
GTTGTGGTAGAGAGTGACAGCGAGGCGATCCAGTATGTTGCTGGCGGAGACTTGGAGACGATATTGAGCA
ATGGACAGGAGTCTGGGGCTGGAAAGGTTCCTGGCAAGTTCTTTTGAGCGGTGTCTGTATGCTTTGTACC
CTATTCTTATCATCACGGCGCAATGGCCCTGGAGATGCATGGACGGCATGCTTACAATGACCACCCAGTC
ATCCCTTATCGGGGTGAACGGCGCAAATGGCTAACGACAAAAAGGGGGCTGGGACAGTTGAGGTAAGGGG
GGCTCAAGCTGTGGAGGTTGGAGGTTGAAGGATTAGTGCGAGGTATTGGCGATGGATAATATGGGGGTCG
AGTTTTGGGTGAGCTTCAGTTTCGATTCTCTTTGTCGTCACACGACGACGACTGCATCACGGAAGCAATC
ATAATAGATGCTGTTACTACACTCATTGAATCAAATCGACACATTCGCTCACCACCATTTGTTTCAAAGA
TTTCCTATCTTGAAATAGGACGTACTATTTCCAG

Description:

In the sequence, the region highlighted with grey indicates the exons and region highlighted with yellow denotes the TPP riboswitch aptamer. Splice sites GT and AG are highlighted in red and viable alternative 5' splice sites GT are highlighted in cyan. Section highlighted in teal represents the α and α′ regions. In the riboswitch aptamer underline sections represent the P1 and P1' domain. In the Type II splicing, TPP riboswitch is resides in a long intron having a length of 650-900 nucleotides and involved in α-α′ base-pairing.

TPP riboswitch mediated Type III Splicing

Figure S4-5 (TPP riboswitches in intron of THI4 gene)

```
>NW_008751647.1:3213964-3215505 Exophiala_dermatitidis THI4
GTTTCTTCTGAACCTTGCCATCAAAGCAAAGCTATCAGTCAGTTTCTTGCCTCAGCATCCCCTAGACAAA
CCCTCCAAATCCACAAACTTTTGTAGTCATCCTCTACCGCATCTGGTTGTCCCCAGCGCTAATCTTCTCC
AGGTCCAAATCGATCGAAAGCACCAAGTAAAACTCACTCAAAAAGATGGAGGGCATCATGTCATCCCGTC
GCTCGGTTAAGAGATATCACGGTCAAGGTACTTTTCGACACTGCGGAGTGTCTGAGCATGAACCGGTGTT
CGCATCTTCCTGCTAGAAGTCTCGCCATCCAACCATCTGTCTGCGGAATGGAGATGGCGGGTCTAGATTG
TGGAAGTGCGTTCTGAGATCATACGGTTGAACTTGACTCTGGATAATACCAGCGGAAGGATCATGCTATT
CTCCCTCTTTCCCCAGGTTGCCCACCGGACAACTATCCTAACGAACCTACAGGACTCTCAACATGTCGCC
ACCAATCGCCACGTACAACGAATTCGACGGCGGCTTCACTGCCACCAAAGAGCTGAAGGGAGCTCCTGAG
ACCAAGAAATTGACCCTCAATGCCAACGGCAATGCCACCCACACCAAACTCCTCGATGAGTTTGGCGGCA
AATGGGATGCCTTCAAGTTCGCACCCATCCGCGAGTCCCAGGTCTCTCGAGCCATGACTCGCCGCTACTT
TGCCGACCTTGACCGGTACGCCGAATCGGACGTCGTCATTGTCGGTGCAGGCTCTTGTGGATTGTCCACT
GCCTACACTCTAGCAAAGGCCCGTCCGGACCTCAAGATCGCCATCATCGAGGCGTCCGTCTCTCCCGGCG
GTGGTTGCTGGCTGGGCGGTCAACTCTTCTCGGCCATGGTCTTGCGGAAGCCGGCCGAGGAGTTCTTGAA
CGACATTGGCGTCCCTTACGAAGATGAGGGCAACTACGTCGTCGTCAAGCACGCCGCGTTGTTCATGAGC
ACCTTGATGAGCAAGGTCTTGGCCATGCCCAATGTCAAGCTCTTCAACGCCACCTGCGTCGAGGATCTCG
TCACTCGTCCATCCGCAGATGGTGGAGTCCGCGTGGTTGGTGTGGTCACCAACTGGACTCTTGTCACTCT
GCACCATGACAACCACAGCTGCATGGATCCCAAGTAAGTTGTGGTCATGAATGTTACTGTGCGTTTATTA
CCTGTACTGACGATTTGTTCCTTGTAGCACCATCAATGCTCCACTGGTCATCAGCACCACCGGCCACGAC
GGTCCATTTGGAGCATTCTGTGCCAAGCGTCTCGTCAGCATGAACGCCATTGAGAAATTGGGTGGTATGC
GTGCTCTGGACATGAACCGCGCAGAGGATGCCATTGTCAAGGGTACCCGTGAGGTATCTCCTGGTTTGAT
CATGGGAGGCATGGAACTCAGTGAGCTCGACGGAGCCAACCGTATGGGACCTACCTTTGGAGCCATGGTT
CTGAGTGGTGTCAAGGCTGCTGAGGAGGCTCTGAAGGTCTTTGAGACCAGAAAGGCCGAGTGCGCCGAGT
AG
```

Figure S4-6 (TPP riboswitches in an intron of NMT1 gene)

```
>NT_166519.1:2566725-2568547 Aspergillus_niger NMT1
CCCATCTTCTCACTATCTATCTTCAGTTATATTGTTTCCGAATAACTTACTTCTTCTTCCCCAACAACTT
CCTCGTTAACCGTCCGGTACGACTCACAATGAGGCCGCGCACGCAGGATCAGACTCCGGGTCTTTTCGCT
ATGGGTTCAGATGGGTCAAGGTATCGTACAATAGTATAACAGTCACTGCTCGCGCATGACAGGTGTTCGG
CTCGTGCTTCTGTTCCTTTCCTTCTGGTTTGGACAGGAGCGCGGTCGTTCTGAGATTATACTGTCAAAAC
TTGATCTAGATAATACTAGCGAAAGGACATGCGTGGCACTGATTGTCCCCTACTATTTGACCTACAGAAG
ACGAGAGGGATCTCGCATCCCTCTCTGTTGCTGACAGTTTCCAGACCTTTGCAATTACCCTCGACCTGAG
TGTATTTGTGCCAAAATGTCTACTGACAAGATCACCTTCCTGACCAACTGGTATGGATTGGGATCTCTAT
TTTGATTTTTAGAAACAGGTCACAGAATATATGCTAAATCATGCGTTCTCTTAAGGCACGCGACCCCGTA
TCACGCCCCCCTATACCTCGCCCAGAGCAAGGGCTACTTCAAGGAAGAGGGCCTTAAAGTTGCTCTCCTG
GAGCCCAATGACCCCTCCGATGTCACCGAGATCATCGGCAGCGGCAAAGTTGACATGGGCTTCAAGGCTA
TGATCCATACCCTTGCTGTATGAGCTACCTTGCCTCTCCCCCGTTCTTCAAATCCTATACAACCAGCATC
TAACCAAAACAACAGGCCAAGGCCCGCAACTTCCCCGTCACCTCGATTGGTTCTCTCCTCGACGAGCCCT
TCACTGGCGTCGTCTACCTCAAGGACAGCGGAATCACCGAAGACTTCCGCTCCCTGAAGGGCAAGAAGAT
```

CGGCTACGTCGGCGAATTCGGCAAG**GT**TCGCCTTCCCTCCTCCCTTCCATTCCACCACCATAACCTAACC
AACCCACTCCAC**AG**ATCCAAATCGACGAGCTCACCAAATACTACGGCATGACCGCCGACGACTACACCGC
CGTCCGCTGCGGCATGAACGTAACCAAAGCCATCATCCGCGGCGACATCGACGCAGGCATTGGCCTCGAG
AACGTGCAGATGGTCGAGCTCGCCGAGTGGCTCGCCACGCAGGGCCGACCCCGCGACGACGTGCAAATGC
TGCGCATCGACCAGCTCGCCGAGCTCGGATGCTGCTGCTTCTGCTCGATCCTGTACATCGCCAACGACGC
CTTCCTGGCCGCCAACCCGGAGAAAGTGCAGAAGTTCATGCGGGCTGTCAAGCGCGCTACGGACTACGTT
CTGGCTGAACCCGCCCGCGCTTTCGAGGAGTACGTTGACATGAAGCCGATCATGGGCACTCCGGTTAACC
GGAAGATCTTTGAGCGTTCGTTCGCGTACTTCAGTCGTGACTTGAAGAATGTTAGCCGCGACTGGGCTAA
GGTGACCAACTATGGCAAGCGGTTGGGCATTCTGAGTGCTGATTTCGTGGCGAACTACACTAACGACTAT
TTGTCGTGGGGCTTGGATGCTGACTCGACTGATCCCCTGGGGGATCAGAAGCGCATGGCTGAGTTGCAGA
AGAAGGTCGCTGCCGAGGGTGGTTACAAGCGGTTGGAGGTTGCTTCTTCGGCTTGATTGATTATCAGCTC
GGTGGAGCAGCTTGCTCGCTTGACACTCTAGGAACAGGGATGATTCTTATGTAACGGTCGTTTACGAGTC
TAGCTGGATGTGCTAGCTTACGCACTCTTTTAGTATGACAGCAATATATCATACGATCTTTTGAGACATG
AAA

Figure S4-7 (TPP riboswitches in an intron of transporter gene)

>NW_001939257.1:613102-614997 Pyrenophora_tritici-repentis nucleoside transporter
AGTCAGTTGACGATTCTCCGCTTGTCGTCGTCATTGTCCATCTCAAGTACG**GT**AACACAGCCACACAGCC
AAAATGCAGCTGAGTACCAGGAAGCCACCCGATCAAGGTAAC**GT**CTGAGCAATATGCATGAGACCG**GTGT**
TCGCCCGTCATCATTCCTGATGGCGGCGATCTGAGATATACGGTCTGAACTTGATCAGGTTAAAGCCTGC
GAAAGGACTCATGCTTTGCGCCCTTCCCCCAAGGCTGCTGCTAATGGGTGT**AG**GTCGCTGTCATGGTAGA
ATTCTGCACGAACAACACCCACGATGACGAGACCAAAGTTGCGCCGAATGCGAGCAATGAACTGCAACTA
CAACTGCCTATCAGCACGCGTGATGCTGGAATCGACGAGCGCTTACGAAATGGGGGATGGCAAGATACGT
TCAAGGCATTCGAGAGGCAGCTCGTGGAATACAACCTCGAGGCGCGTGGCATTCAGCGTGTAGAGCCAGA
CGAGCGACAAGACTTGCGCCTCCTGGGATACTCGCAGGTCGCTATCATGTGGTTTAGCGTCAACCTGGCT
GCAAACAACATCACCCTAGGTATGCTGGGCCCCGCTGTCTTCGCGCTCGGATTCCTTGACTCCTGTCTGC
TGGCCGTCTTTGGCGTATCTGTGGGCTGTCTTGTTGTCGCATACATTGCCACCTTTGGGCCGAAGAGTGG
AAATCGGACCATGGTATTCACACGCTATGTCACAGGATGGTGGCCGTCTAGAATTGTTGTCTTACTCAAT
ATTGTCGTCCTTTTGGGTTACGGCATGATCGACTGCGTAGTAGCCGGACAGATACTGTCTGCCGTCTCTG
GAAATTCCATGTCTGTCGTAGTCGGCATCATCATCGTGGCCGTGATTGCATGGATCATCACAACGTTCGG
ATATCGAATATTCCACTATTATGAGCGCTGGGCGTGGCTCCCCCAGTTAATTGTGCTCTCCGTGTTAGCT
GGTATCGCAGGGCCACACTTCAACATCTCTTCCCAGTCGACTGGCGATACGGATCCGGACACCATAATAG
GAAACCGCATTAGCTTTTTCGGCCTGACACTAGCTGCGGCTATCACATACGGTGGTGGCGCGGCTGATTA
TTTTGTTTACTATCCCGAACACTCATCGTCCTTGAAAATCTTCGCAATGACCATGCTAGGTCTTATGTGC
AGCTTTACATTTGCCTTTATCTTAGGTATCGGCCTAGCATCCGGCATGTCAACCAATAGCAACTGGGAAG
CAGCATACGGTGTATCCCAGGGCGCTCTCATTGTCGAAGCATACAAGCCTCTTGGCGGGTTTGGTTCTTT
CTGTGGTGTCATCGTCGCTCTTGGTCTTGTCGCCAATCTCATTCTTCCCACATACTCTTCTGGTATTGAT
GCCCAGATCCTTGGTCGGTACGCAGGCGCGATACCGAGAGTAATCTGGAACACCATCGGCGTTGTCATAT
ACACAATATGTGCATTAGCCGGACGTGCTCATCTTGCCGAGATCTTTACCAATTTCCTGGCTCTGATGGG
TTACTGGGTATCTGTCTGGGTCGCTATCGTTCTAGAAGAGCATTTGATCTTTCATCGCCAGGCAGGCTTT
AATTGGGGAGTTTGGAATCAAAAGAAGAAGCTCCCTCTGGGTATTGCAGCATTTGCAGCCTTTGTCATCG
GTTGGATAGGCGCTATCATGTGCATGGCACAAGTTTGGTATATCGGGCCAATCGCCAAGCAAGTTGGTAC
CCACGGAGGAGAT**GT**AAGTCTCTTTAACATTGTAGCTGTTGATATTTAGCTGATTAGTCT**AG**ATGGGCAA
TTTTATTGGCTTTGCATGGGCGGCAGTTGTATATCCTCCCCTAAGGATATTGGAACTCAAGCGGTTCGGA
AGATGA

Description:

In these sequences, the region highlighted with grey indicates the exons and region highlighted with yellow denotes the TPP riboswitch. Splice sites GT and AG are highlighted in red, internal stop codon is highlighted in green and viable alternative 5' splice sites GT are highlighted in cyan. In the Type II splicing, TPP riboswitches located in an intron having length 200-400 nucleotides.

Figure S5

Figure S5: Consensus structures of Type I, II, III and IV fungal TPP riboswitches.

Figure S5-1: Consensus structure of fungal Type I TPP riboswitch

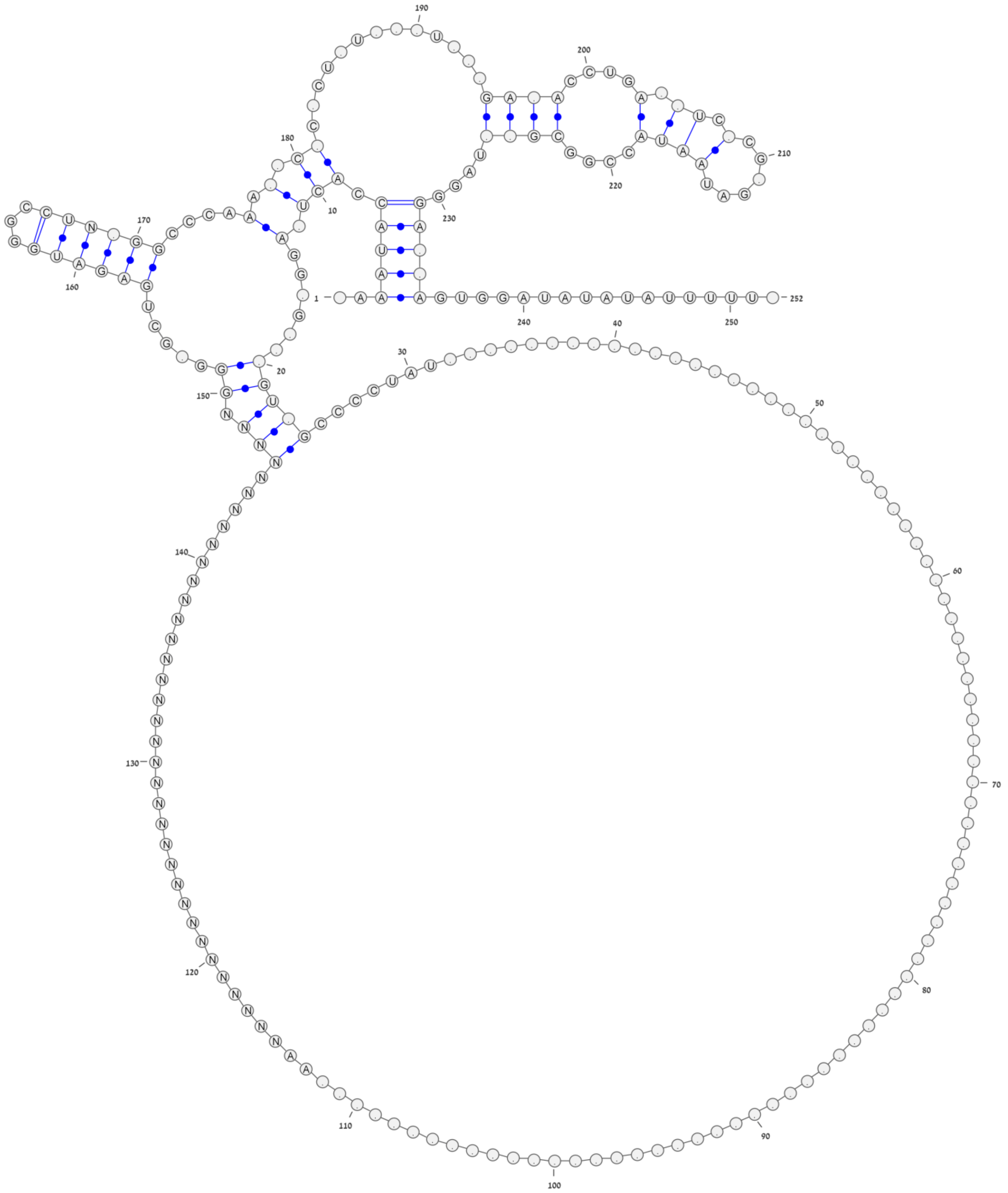

Type i

Figure S5-2: Consensus structure of fungal Type II TPP riboswitch

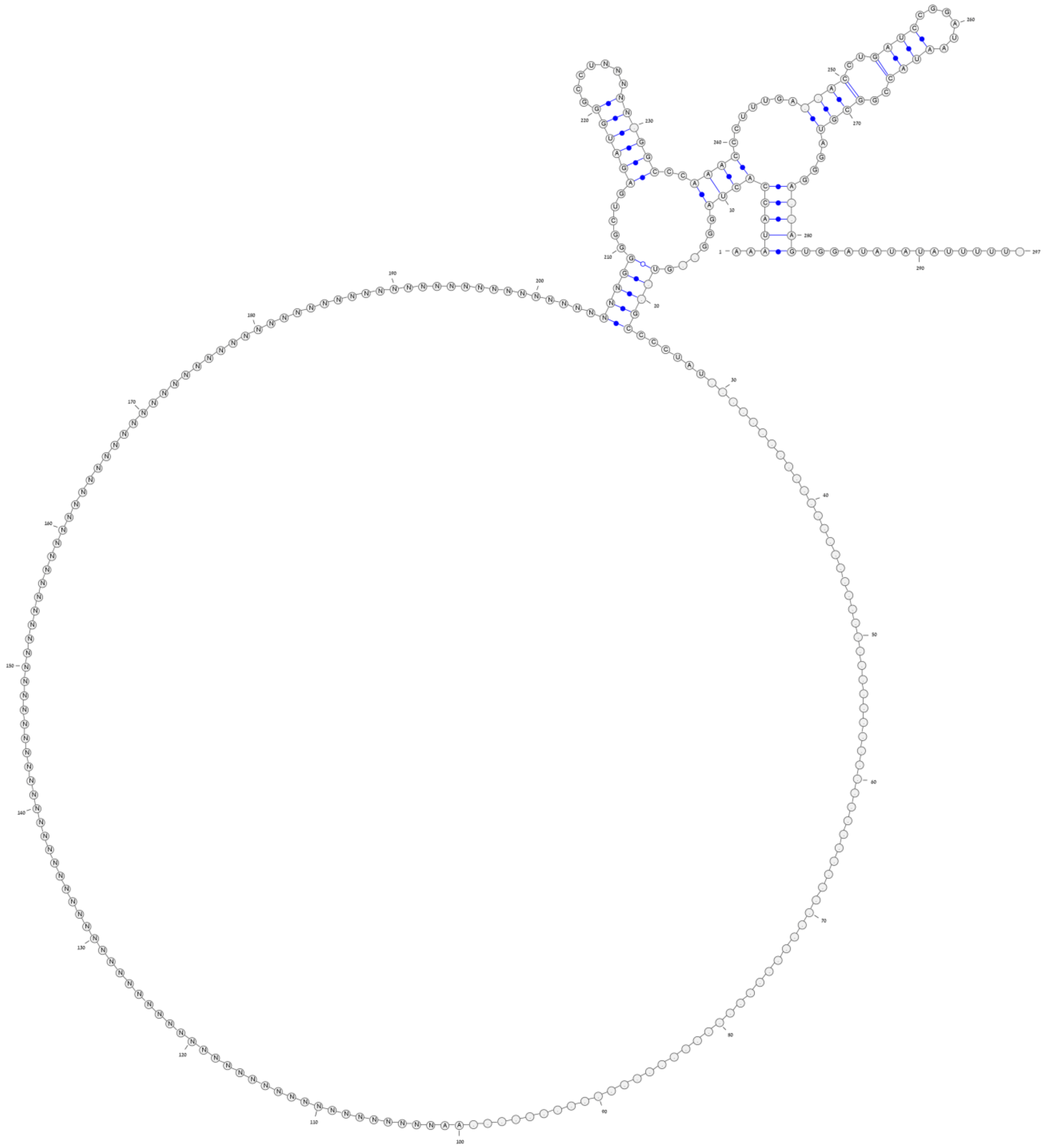

Type ii

Figure S5-3: Consensus structure of fungal Type III TPP riboswitch

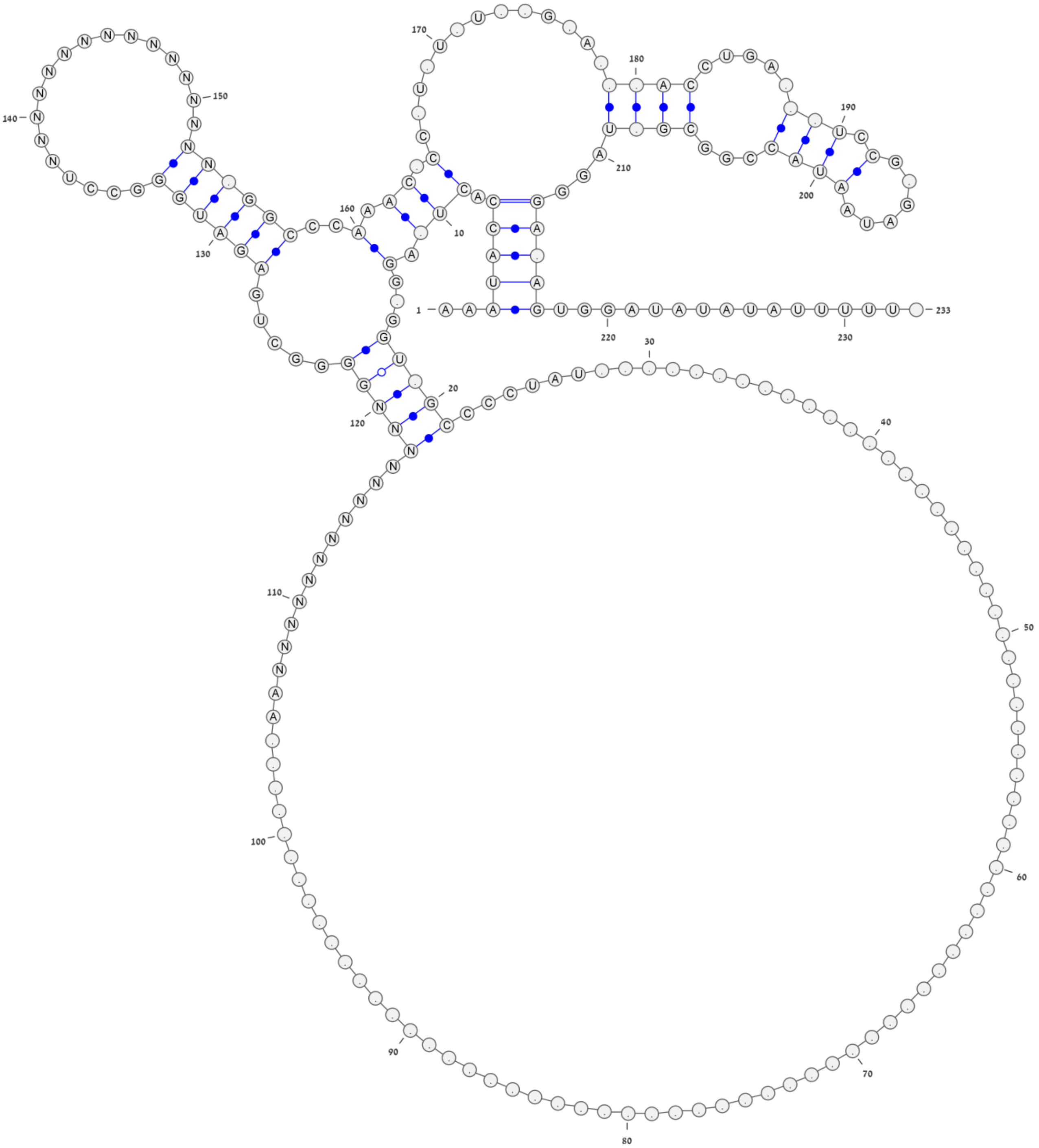

Type iii

Figure S5-4: Consensus structure of fungal Type IV TPP riboswitch

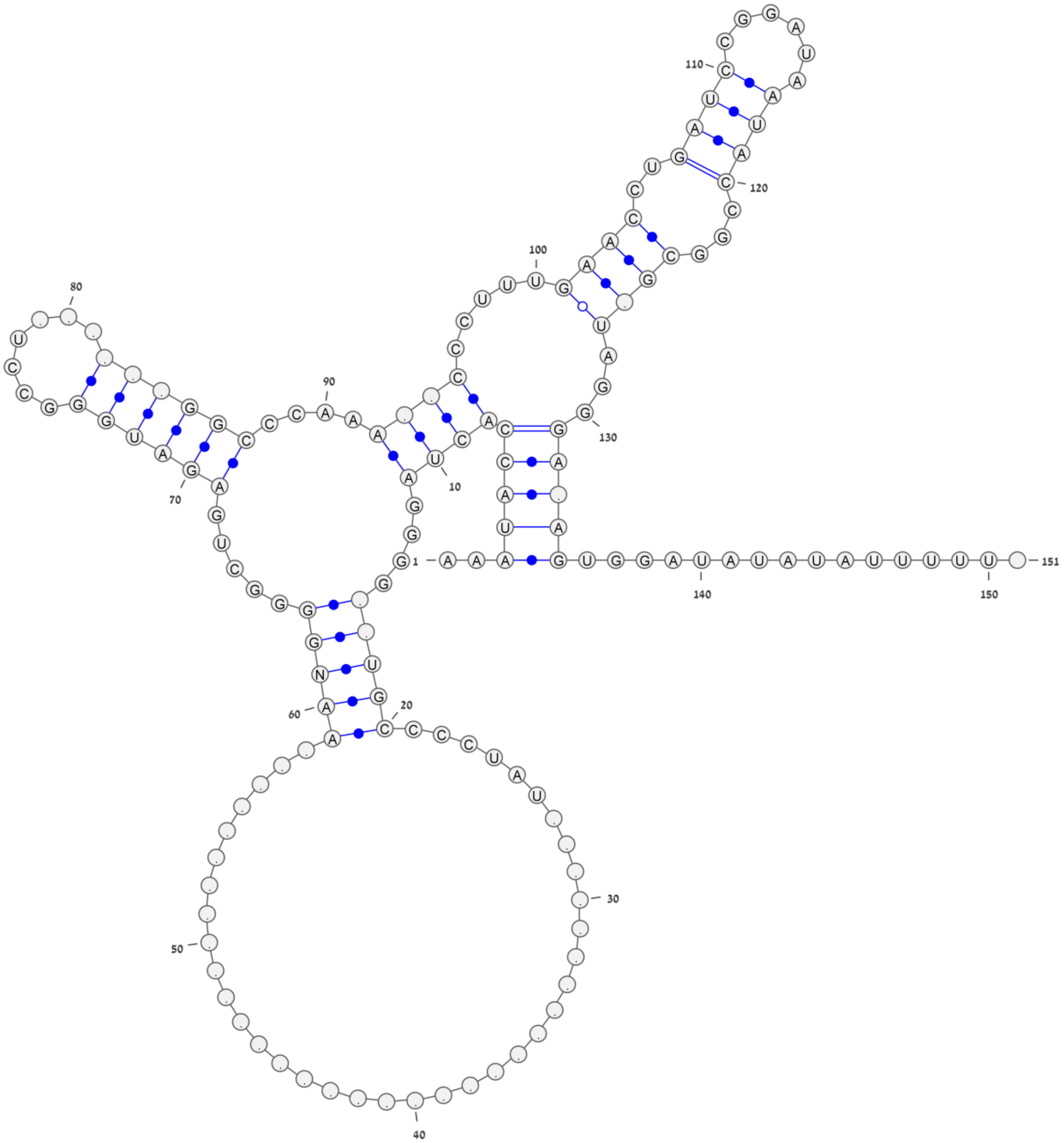

Type iv

Figure S6

Figure S6: Validation of the phylogenetic marker genes. The phylogenetic trees, generated from the two random sets of species distributed across three fungal phyla and constructed using a combination of the four marker genes, are depicted. In both cases, the four marker genes selected are able to accurately classify the fungal phylogeny producing well-resolved trees.

Figure S6-1
Fungal tree of life - Set 1

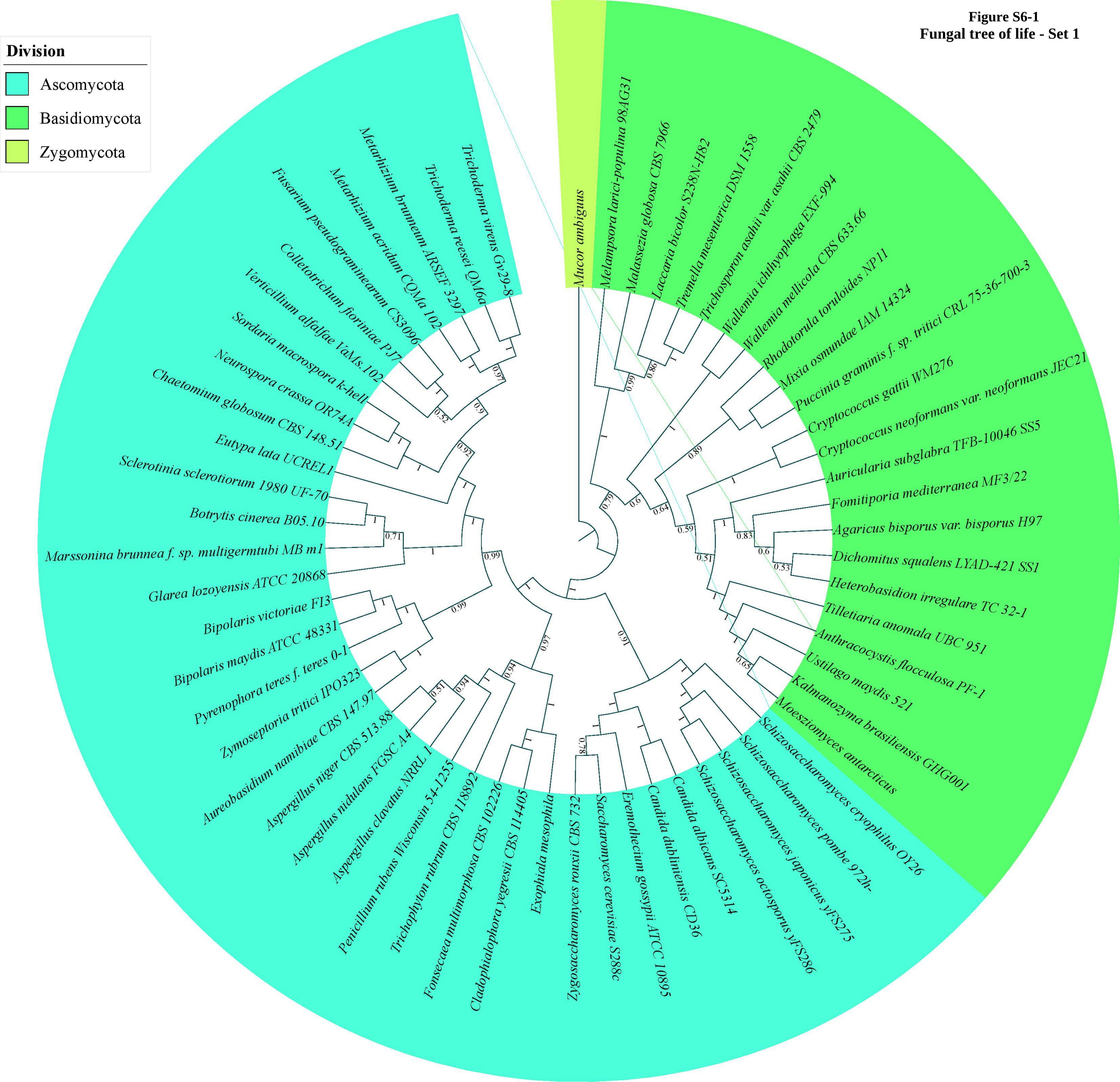

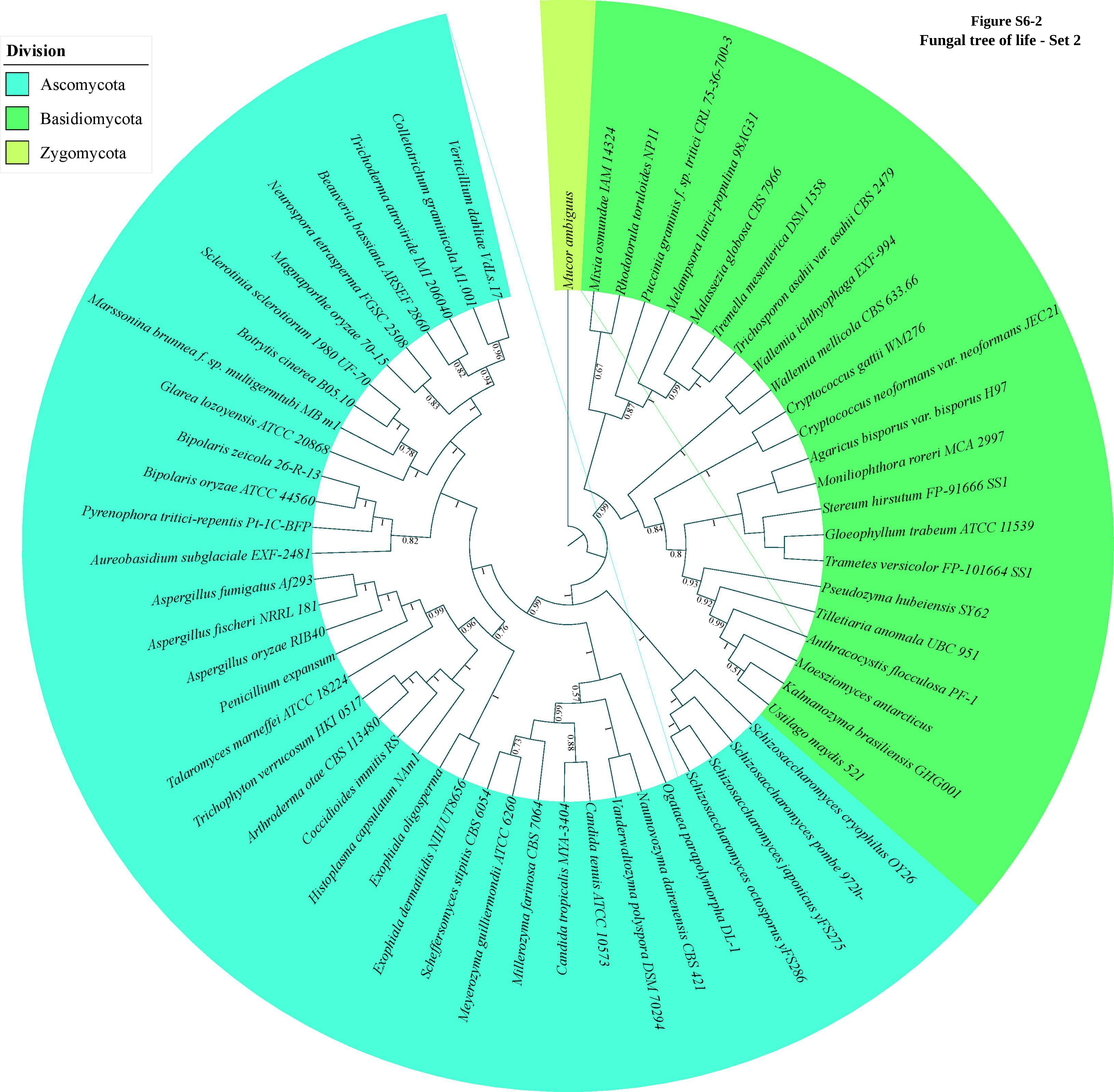

Figure S6-2
Fungal tree of life - Set 2
